# Real‐Time Visualization of Isoform‐Specific RAF‐KRAS Interactions in Living Cells Using FRET‐BRET Hybrid Biosensors

**DOI:** 10.1002/advs.202515654

**Published:** 2026-02-12

**Authors:** Jeong‐Min Go, Dahee Lee, Minji Kim, Kiseok Han, Gyuho Choi, ChanHui Song, Sanghyun Ahn, Yerim Lee, Jinyoung Lee, Yingxiao Wang, Jung‐Soo Suh, Hwayoung Yun, Tae‐Jin Kim

**Affiliations:** ^1^ Department of Integrated Biological Science College of Natural Sciences Pusan National University Busan Republic of Korea; ^2^ College of Pharmacy and Research Institute for Drug Development Pusan National University Busan Republic of Korea; ^3^ Alfred E. Mann Department of Biomedical Engineering University of Southern California Los Angeles California USA; ^4^ Department of Biological Science, and Institute of Systems Biology College of Natural Sciences Pusan National University Busan Republic of Korea

**Keywords:** biosensor, BRET, FRET, KRAS, live‐cell imaging, RAF

## Abstract

The RAS–RAF–MEK–ERK signaling cascade is a central component of the mitogen‐activated protein kinase (MAPK) pathway, regulating cell proliferation, differentiation, and survival, and is frequently dysregulated in cancer. Despite extensive biochemical characterization, direct observation of KRAS interactions with RAF isoforms in living cells remains limited. To overcome this limitation, a dual‐mode biosensor platform is presented that enables real‐time monitoring of RAF‐KRAS interactions through both fluorescence resonance energy transfer (FRET) and bioluminescence resonance energy transfer (BRET). Isoform‐specific biosensors reveal distinct interaction dynamics, with ARAF‐based sensors exhibiting the strongest and most reversible FRET responses. Importantly, incorporation of NanoLuc luciferase into the hybrid biosensor preserves FRET sensitivity while introducing a luminescent BRET mode suitable for high‐throughput and low‐background applications. Evaluation of oncogenic KRAS mutants indicates elevated basal FRET signals and differential binding profiles across RAF isoforms. Pharmacologic profiling further demonstrates allele‐selective inhibition, with mutant‐specific FRET and BRET responses observed upon treatment with targeted KRAS inhibitors. This biosensor platform enables live‐cell, real‐time, and quantitative monitoring of RAF‐KRAS interactions, facilitating the analysis of oncogenic signaling dynamics and the evaluation of mutation‐specific responses to targeted therapies under physiologically relevant conditions.

## Graphical abstract

Hybrid FRET–BRET biosensors were developed to monitor RAF–KRAS interactions in living cells. These sensors enable real‐time visualization of interaction dynamics, quantitative spectral analysis, and validation of mutation‐selective inhibitors, providing a versatile platform for probing KRAS regulation and therapeutic responses.

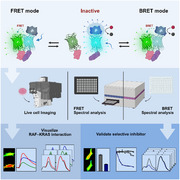

## 1 Introduction

The RAS–RAF–MEK–ERK signaling cascade represents a central axis of the mitogen‐activated protein kinase (MAPK) pathway, regulating cell proliferation, differentiation, and survival [1, 2]. At the top of this cascade, the interaction between RAS and RAF functions as a critical molecular switch, linking receptor tyrosine kinase activation to downstream signaling. Among the three RAS isoforms–KRAS, NRAS, and HRAS–KRAS exhibits the strongest association with human cancers, playing a pivotal role in tumor initiation and maintenance across a wide range of malignancies [3, 4]. Epidemiologic and genomic studies have shown that KRAS mutations occur in approximately 30 % of all solid tumors, with the highest prevalence in pancreatic cancer (up to 90 %), colorectal cancer (40 %–50 %), and non‐small cell lung cancer (25 %–30 %) [5, 6, 7, 8]. These mutations are most commonly found at glycine 12 (G12), a hotspot within the P‐loop region of KRAS that is critical for GTP binding and hydrolysis, and that gives rise to distinct mutant forms, including G12C, G12D, and G12V, whose distribution varies across tumor types. KRAS mutations impair the intrinsic GTPase activity of the protein, rendering it resistant to inactivation by GTPase‐activating proteins (GAPs) [9]. As a result, mutant KRAS remains in a constitutively active GTP‐bound state, persistently recruiting downstream effectors such as RAF kinases to propagate oncogenic signaling. Despite the central role of RAF‐KRAS interactions in driving malignancy, the real‐time dynamics in living cells remain poorly understood, in part due to the lack of tools capable of resolving isoform‐specific and mutation‐sensitive binding kinetics. While in vitro biochemical approaches such as isothermal titration calorimetry (ITC), surface plasmon resonance (SPR), and pulldown assays have revealed that BRAF‐KRAS interactions are generally stronger than those of ARAF or CRAF, these measurements lack the temporal resolution and regulatory context necessary to capture dynamic events in intact cells [10, 11, 12].

Fluorescence‐based biosensors, particularly Förster resonance energy transfer (FRET) systems, have been instrumental in visualizing protein‐protein interactions in live cells [13, 14]. These sensors report molecular proximity via energy transfer between two fluorophores, typically within ∼10 nm, and offer high spatial and temporal resolution [15, 16]. However, conventional FRET biosensors are often constrained by photobleaching, autofluorescence, and the requirement for external excitation light, which limits their sensitivity and long‐term imaging capability [17, 18]. Alternatively, bioluminescence resonance energy transfer (BRET) systems utilize luciferase enzymes as donors, providing excitation‐independent and background‐free detection [19, 20]. Nevertheless, BRET suffers from lower photon yield and short substrate half‐life, restricting its dynamic range and multiplexing potential [21]. To address these limitations, hybrid biosensors that incorporate both FRET and BRET functionalities have emerged as a promising strategy [18]. Recent studies have demonstrated the feasibility of such dual‐mode sensors in capturing diverse signaling events while maintaining compatibility with both imaging and plate‐based assays. Yet, to our knowledge, no biosensor platform has been developed that can simultaneously resolve KRAS mutation effects, RAF isoform‐selectivity, and pharmacologic inhibition in live cells through dual‐mode detection.

In this study, we report the development of a genetically encoded RAF‐KRAS interaction biosensor platform capable of dual‐mode analysis via both FRET and BRET modalities. This hybrid biosensor system allows real‐time monitoring of RAF‐KRAS binding dynamics with isoform specificity and mutation sensitivity, overcoming previous technical constraints of intensiometric or single‐mode systems. We applied this platform to dissect RAF isoform‐dependent binding kinetics, visualize oncogenic KRAS mutant behaviors, and evaluate mutation‐selective inhibition by small molecule compounds such as sotorasib and MRTX1133. Our findings not only provide mechanistic insights into RAF‐KRAS complex regulation but also demonstrate the potential of this hybrid biosensor platform as a versatile screening tool for targeted RAS therapeutics.

## 2 Results

### 2.1 Design and Development of a FRET‐Based RAF‐KRAS Biosensor

**FIGURE 1:**
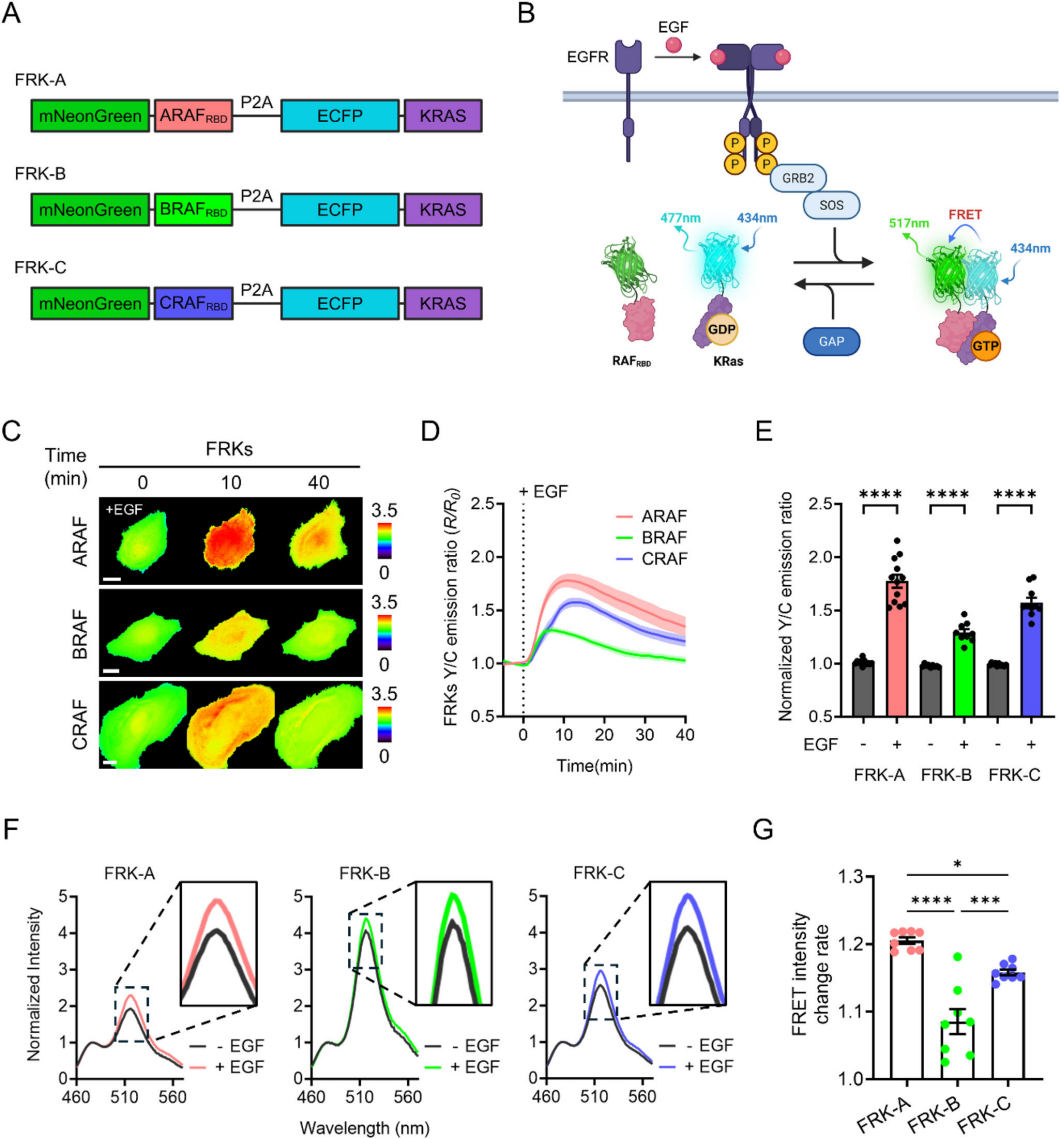
Genetically encoded FRET biosensors visualize RAF‐KRAS interactions in living cells. (A) Schematic diagram of FRK‐A, FRK‐B, and FRK‐C biosensors. (B) Model illustrating how FRKs detect RAF‐KRAS interactions based on KRAS nucleotide state and RAF‐RBD binding. (C) Representative time‐lapse FRET/CFP ratio images of HeLa cells expressing FRK‐A, FRK‐B, or FRK‐C following stimulation with 50 ng/mL EGF. Regions with higher FRET efficiency are visualized in warm colors, while lower FRET efficiency appears in cool colors based on the FRET/CFP ratio. Scale bar = 20 µm. (D) Time‐course of the mean normalized FRET/CFP ratio changes in FRK‐A (n = 12), FRK‐B (n = 9), and FRK‐C (n = 10) before and after treatment with 50 ng/mL EGF. (E) Peak FRET/CFP ratio quantified from individual traces in (D), representing the maximal EGF‐induced response for each FRK variant. Each dot indicates a single cell (^****^ *p* < 0.0001). (F) Normalized FRET emission spectra of FRK‐A (n = 8), FRK‐B (n = 8), and FRK‐C (n = 8) in Lenti‐X 293T cells treated with 50 ng/mL EGF or DMSO (0.5 % v/v, control) for 10 min. (G) Quantification of the EGF‐induced increase in FRET emission at 517 nm, expressed as the post‐/pre‐treatment intensity ratio from (F). Bars represent the fold changes for each FRK variant; each dot indicates an individual spectrum (^*^ *p* < 0.05, ^***^ *p* < 0.001, ^****^ *p* < 0.0001). Data are shown as mean ± SEM. Statistical significance was determined using a two‐tailed unpaired *t*‐test or one‐way ANOVA with Tukey's post hoc test.

To facilitate real‐time monitoring of isoform‐specific RAF‐KRAS interactions in living cells, a set of genetically encoded FRET‐based RAF‐KRAS biosensors, termed FRKs, was developed. Each construct consists of ECFP and mNeonGreen, functioning as a FRET donor–acceptor pair, connected via a P2A sequence to ensure independent translation of each protein component. The sensing module comprises the RAS‐binding domain (RBD) from one of the three RAF isoforms, ARAF, BRAF, or CRAF, and full‐length KRAS expressed as separate P2A‐linked components, enabling dynamic detection of RAF‐KRAS binding events specific to each isoform (Figure 1A). The sensor mechanism relies on conformational proximity changes upon KRAS activation. Upon stimulation with epidermal growth factor (EGF), KRAS undergoes guanine nucleotide exchange via upstream GEFs, transitioning from its inactive GDP‐bound state to an active GTP‐bound form [22, 23]. Activated KRAS subsequently interacts with RAF‐RBD [24, 25], bringing the two fluorophores into close proximity and inducing FRET (Figure 1B). This configuration enables sensitive detection of RAF‐KRAS binding downstream of receptor tyrosine kinase activation, with high spatial and temporal resolution.

Following stimulation with 50 ng/mL EGF, an increase in FRET channel intensity was observed at the plasma membrane in cells expressing each of the FRKs, as visualized by fluorescence microscopy (Figure S1A,C,E). This observation is consistent with previous findings that activated KRAS binds to RAF at the plasma membrane [26, 27]. Approximately 10 min after EGF stimulation, the FRET signal reached its peak while the ECFP intensity reached its minimum, confirming that energy transfer was occurring efficiently between the fluorophores (Figure S1B,D,F).

Subsequent time‐lapse analysis of FRET/CFP ratios revealed isoform‐specific signaling kinetics. FRK‐A (ARAF) showed the most rapid and robust increase in FRET ratio, reaching peak values at approximately 10 min, while FRK‐B (BRAF) and FRK‐C (CRAF) peaked at 8 and 13 min, respectively (Figure 1C,D). Notably, the ratio increase was distributed across both the membrane and cytoplasmic regions, indicating that the biosensors reliably capture global RAF‐KRAS association dynamics. Quantitative comparison of FRET ratio fold changes demonstrated that FRK‐A exhibited the highest response amplitude among the three variants, suggesting its superior sensitivity to KRAS activation (Figure 1E). These isoform‐dependent differences may reflect variations in binding affinity or regulatory feedback among RAF paralogs [10, 28]. To further validate these results, the emission spectra were recorded under the same stimulation conditions. Upon excitation at 434 nm, all biosensors exhibited enhanced emission at 517 nm following EGF stimulation, with FRK‐A showing the largest increase (Figure 1F). Spectral quantification supported this finding, with FRK‐A yielding the strongest EGF‐induced shift relative to baseline (Figure 1G).

Because expression of RBD‐containing sensors can, in principle, perturb endogenous MAPK signaling, we next evaluated whether FRK expression alters downstream ERK activity. ERK signaling was monitored using an ERK kinase translocation reporter (ERKKTR‐mRuby2) in mock‐transfected and FRK‐A‐expressing cells under both basal and 50 ng/mL EGF‐stimulated conditions (Figure S2A–D) [29]. ERK KTR responses were comparable between the two conditions, indicating that FRK expression does not measurably interfere with endogenous ERK signaling in this experimental context.

Collectively, these results confirm that the FRKs reliably report KRAS activation and subsequent RAF binding in live cells, establishing FRK‐A as the most dynamically responsive and sensitive construct among the isoform‐specific variants.

### 2.2 Live‐Cell Monitoring of Reversible RAF‐KRAS Interaction

KRAS cycles between its active GTP‐bound and inactive GDP‐bound states, a process tightly regulated by guanine nucleotide exchange factors (GEFs) and GTPase‐activating proteins (GAPs) [30]. GEFs promote KRAS activation by facilitating GDP‐GTP exchange in response to upstream signals such as EGF, whereas GAPs promote inactivation by accelerating GTP hydrolysis. Previous studies have shown that the interaction between RAF and KRAS peaks approximately 10 min after EGF stimulation, reflecting a transient activation event [31].

**FIGURE 2:**
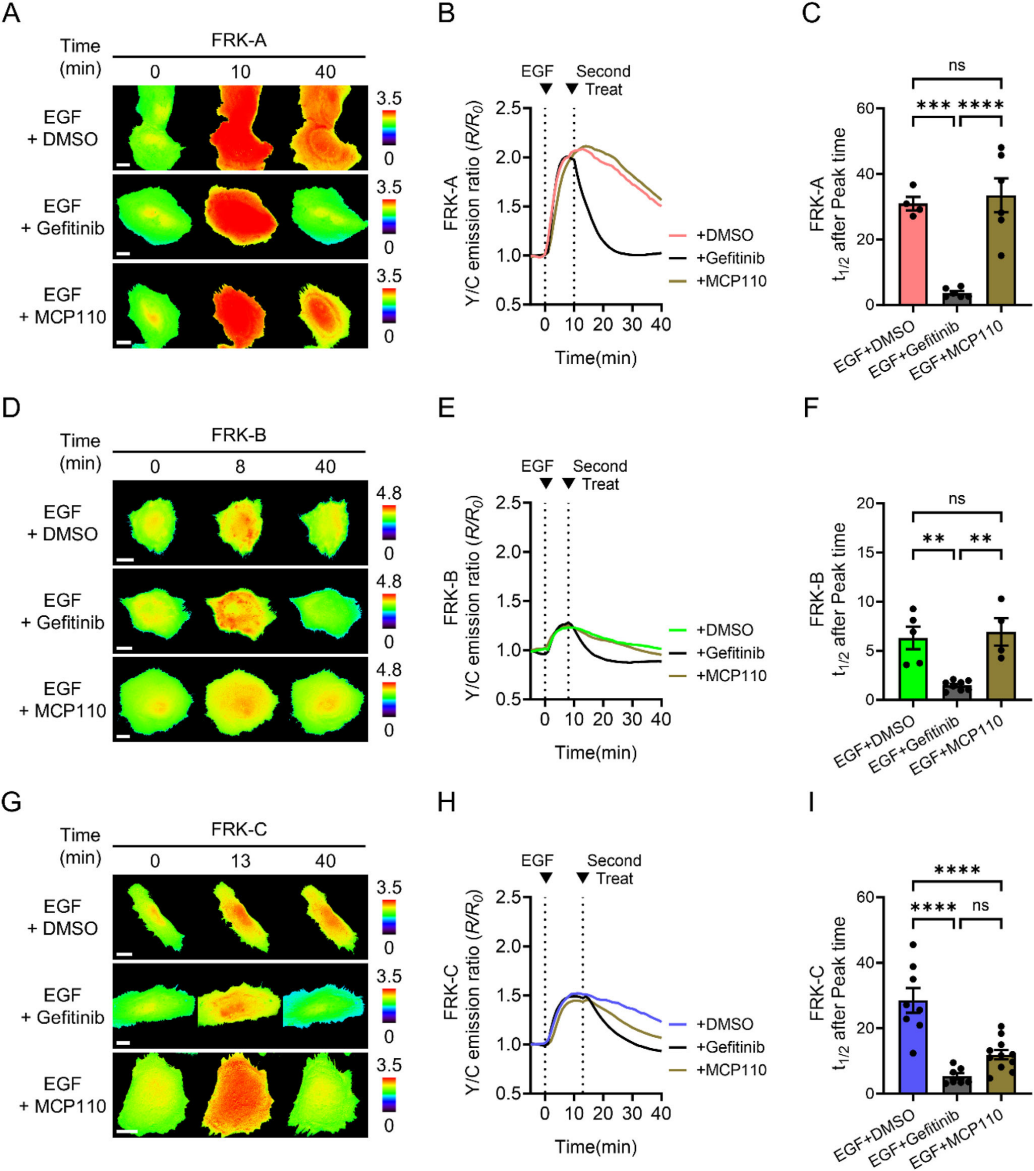
Real‐time monitoring of RAF‐KRAS dissociation dynamics using FRK biosensors. (A) Representative time‐lapse FRET/CFP ratio images of HeLa cells expressing FRK‐A. Scale bar = 20 µm. (B) Time‐course curves and (C) quantification of FRET signal decay kinetics. Cells were stimulated with 50 ng/mL EGF for 10 min and then treated with 400 nM gefitinib (n = 6), 100 nM MCP110 (n = 6), or DMSO (0.5 % v/v, control) (n = 4). The half‐life (t_1/2_) of the FRET signal decrease was calculated based on the post‐peak decline (^***^ *p* < 0.001, ^****^ *p* < 0.0001, ns: not significant). (D) Representative time‐lapse FRET/CFP ratio images, (E) time‐course curves, and (F) quantification of FRET signal decay kinetics in HeLa cells expressing FRK‐B. Scale bar = 20 µm. Cells were stimulated with 50 ng/mL EGF for 8 min and then treated with 400 nM gefitinib (n = 8), 100 nM MCP110 (n = 4), or DMSO (0.5 % v/v, control) (n = 5) (^**^ *p* < 0.01, ns: not significant). (G) Representative time‐lapse FRET/CFP ratio images, (H) time‐course curves, and (I) quantification of FRET signal decay kinetics in HeLa cells expressing FRK‐C. Scale bar = 20 µm. Cells were stimulated with 50 ng/mL EGF for 13 min and then treated with 400 nM gefitinib (n = 7), 100 nM MCP110 (n = 11), or DMSO (0.5 % v/v, control) (n = 8) (^****^ *p* < 0.0001, ns: not significant). Data are presented as mean ± SEM, and statistical significance was calculated using one‐way ANOVA followed by Tukey's post hoc test.

To assess whether the FRKs could faithfully capture the full temporal dynamics of RAF‐KRAS interactions–including both complex formation and dissociation–in living cells, we conducted a reversible binding assay using time‐controlled inhibitor treatments. Cells expressing FRK‐A, FRK‐B, or FRK‐C were stimulated with 50 ng/mL EGF and subsequently treated with inhibitors at the individual peak response timepoints identified for each sensor: 10 min for FRK‐A, 8 min for FRK‐B, and 13 min for FRK‐C (Figure 2A,D,G). Two inhibitors with distinct mechanisms of action were employed: gefitinib [32], an EGFR tyrosine kinase inhibitor that blocks upstream signaling, thereby preventing further KRAS activation; and MCP110 [33], a small molecule known to specifically disrupt the KRAS‐CRAF interaction.

In cells expressing FRK‐A and FRK‐B, gefitinib treatment induced a significantly more rapid decline in the FRET/CFP ratio compared to DMSO controls, indicating effective cessation of KRAS activation and subsequent dissociation of RAF‐KRAS complexes (Figure 2A–F). Conversely, MCP110 had no appreciable effect on the FRET signal in these two constructs, consistent with their lack of CRAF‐RBD incorporation and supporting the specificity of MCP110 for CRAF‐dependent interactions. In contrast, FRK‐C, which incorporates the RBD of CRAF, responded robustly to MCP110 treatment, showing a FRET decline comparable to that of gefitinib (Figure 2G–I). These results indicate that MCP110 selectively disrupts KRAS‐CRAF binding, while gefitinib acts broadly by inhibiting upstream signaling inputs. To exclude the possibility that the observed FRET ratio decreases arose from photobleaching or intensity instability, we next examined the time‐course fluorescence intensities of the donor and acceptor channels during the reversible assay (Figure S3A–F). Neither channel displayed a progressive or monotonic loss of fluorescence characteristic of photobleaching. These observations support the conclusion that the inhibitor‐dependent ratio changes reflect genuine alterations in sensor output rather than artifacts of fluorescence decay.

Taken together, these findings validate the ability of FRK biosensors to dynamically report both assembly and disassembly of isoform‐specific RAF‐KRAS interactions in a live‐cell context, providing a powerful tool for monitoring signal‐induced and inhibitor‐sensitive protein‐protein interaction kinetics.

### 2.3 Development of Hybrid RAF‐KRAS Biosensors

**FIGURE 3:**
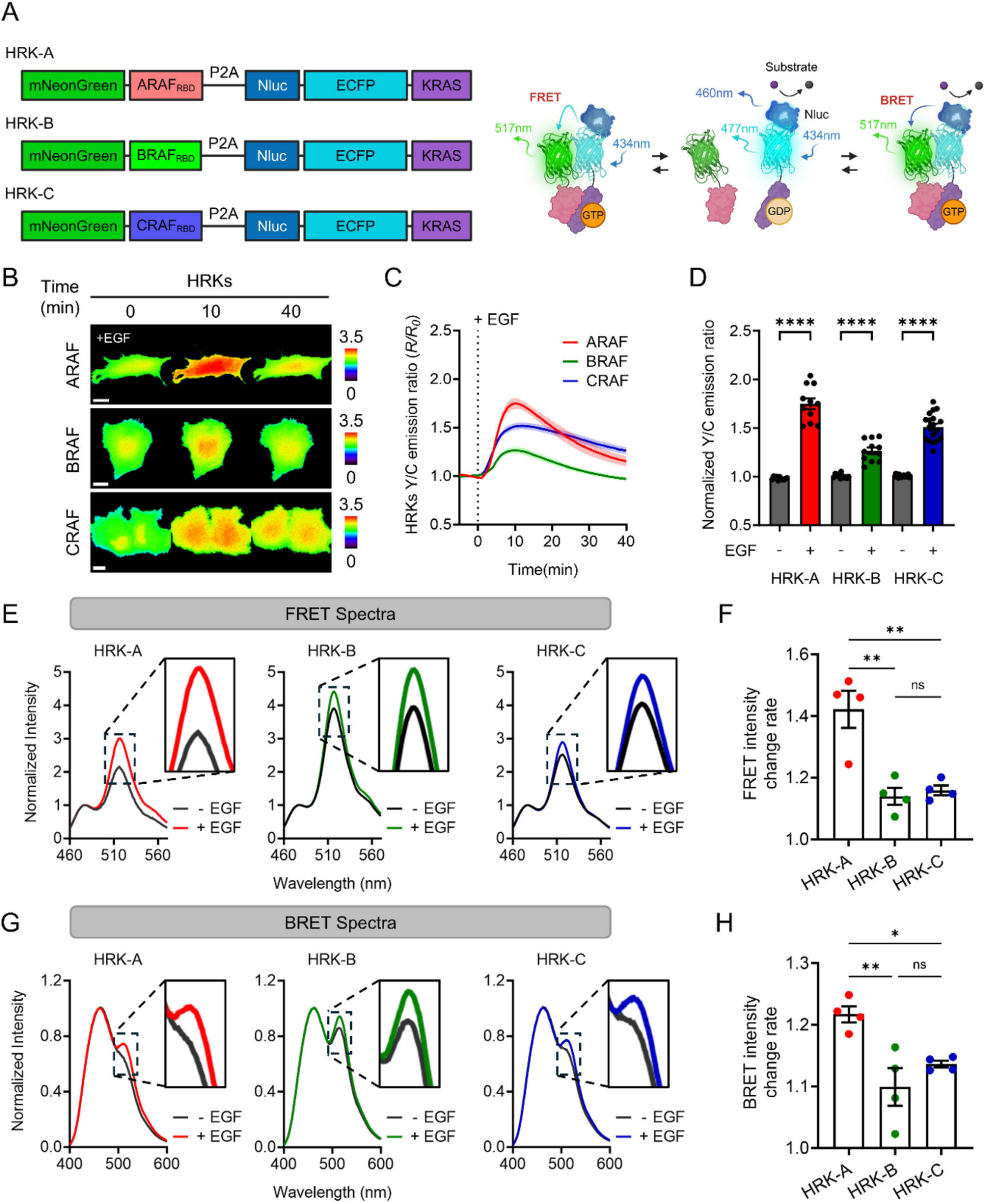
Dual‐mode FRET and BRET visualization of RAF‐KRAS interaction using hybrid biosensors. (A) Schematic diagram of HRK‐A, HRK‐B, and HRK‐C biosensors and the sensing mechanisms. (B) Representative time‐lapse FRET/CFP ratio images of HeLa cells expressing HRK‐A, HRK‐B, or HRK‐C following stimulation with 50 ng/mL EGF. Scale bar = 20 µm. (C) Time‐course of the mean normalized FRET/CFP ratio changes of HRK‐A (n = 11), HRK‐B (n = 11), and HRK‐C (n = 18) before and after treatment with 50 ng/mL EGF. (D) Quantification of the peak FRET/CFP ratio derived from individual traces in (C), representing the maximal response of each HRK variant to EGF stimulation. Each dot indicates a single cell (^****^ *p* < 0.0001). (E) Normalized FRET emission spectra of HRK‐A (n = 4), HRK‐B (n = 4), and HRK‐C (n = 4) in Lenti‐X 293T cells treated with 50 ng/mL EGF or DMSO (0.5 % v/v, control) for 10 min. (F) Quantification of the EGF‐induced increase in FRET emission at 517 nm, calculated as the ratio of intensity after EGF treatment to baseline in (E) (^**^ *p* < 0.01, ns: not significant). (G) Normalized BRET emission spectra of HRK‐A (n = 4), HRK‐B (n = 4), and HRK‐C (n = 4) measured in Lenti‐X 293T cells 10 min after 50 ng/mL EGF or DMSO (0.5 % v/v, control), following Nluc substrate addition. (H) Quantification of the EGF‐induced increase in BRET emission at 517 nm, calculated as the ratio of intensity after EGF treatment to baseline in (G) (^*^ *p* < 0.05, ^**^ *p* < 0.01, ns: not significant). Data are presented as mean ± SEM, and statistical significance was calculated using a two‐tailed unpaired *t*‐test or one‐way ANOVA followed by Tukey's post hoc test.

To expand the utility of RAF‐KRAS biosensors beyond cultured cells, we engineered a hybrid RAF‐KRAS biosensor (HRK) platform that integrates both fluorescence‐ and luminescence‐based detection capabilities. This dual‐mode system was designed to overcome several limitations associated with conventional FRET‐based biosensors–most notably, their reliance on short wavelength external excitation, which limits deep tissue imaging and introduces phototoxicity and autofluorescence [18, 34]. HRKs retain the modular structure of the previously developed FRK sensors while incorporating NanoLuc luciferase (Nluc) as a luminescent donor upstream of ECFP (Figure 3A, left) [35]. Each HRK construct contains mNeonGreen, ECFP, and full‐length KRAS, together with a RAF‐binding domain (RBD) from either ARAF, BRAF, or CRAF, allowing isoform‐specific interaction monitoring. These components are separated by a P2A self‐cleaving sequence to ensure stoichiometric and independent expression of each domain. This architecture allows the same biosensors to report both FRET and BRET signals depending on the detection context, thereby enhancing flexibility and detection range. In the FRET mode, external excitation of ECFP at 434 nm induces energy transfer to mNeonGreen at 517 nm upon RAF‐KRAS complex formation, producing a ratiometric FRET signal. In parallel, the BRET mode utilizes the bioluminescent properties of Nluc, which emits light at ∼460 nm following substrate addition. When KRAS binds to RAF‐RBD, bringing Nluc and mNeonGreen into close proximity, energy transfer from Nluc to mNeonGreen occurs, generating a measurable BRET signal (Figure 3A, right). This dual‐readout configuration allows HRKs to be applied flexibly depending on the experimental context.

To determine whether HRKs retain FRET performance comparable to FRKs, we performed time‐lapse imaging following EGF stimulation (50 ng/mL) and observed dynamic increases in FRET/CFP ratio across all HRK variants (Figure 3B). Among them, HRK‐A exhibited the most pronounced signal increase, followed by HRK‐C and HRK‐B, recapitulating the isoform‐specific response pattern observed with the original FRK sensors (Figure 3C,D). To further assess signal fidelity, we examined the underlying fluorescence channels during EGF stimulation by plotting the time courses of the donor and acceptor intensities (Figure S4A–F). These traces showed stable fluorescence signals over time, supporting the conclusion that the observed ratio dynamics reflect bona fide HRK sensor responses rather than artifacts arising from intensity drift. To confirm that insertion of Nluc did not impair FRET detection [36, 37], we directly compared FRET/CFP ratios between FRK and HRK sensors under both basal and EGF‐stimulated conditions (Figure S5A–F). No significant differences were observed for any isoform, indicating that Nluc integration does not interfere with fluorophore orientation or FRET efficiency. Notably, basal FRET levels differed across RAF isoforms in both sensor formats. BRAF‐based constructs consistently exhibited higher basal FRET ratios, suggesting a greater degree of spontaneous BRAF‐KRAS interaction in the absence of stimulation [28]. In contrast, ARAF‐ and CRAF‐based sensors showed relatively low basal FRET, reflecting tighter regulation or lower intrinsic affinity.

To evaluate RAF‐KRAS interactions at the population level, we conducted spectral analysis using both FRET and BRET detection modes. In FRET‐based spectra, all HRK variants exhibited increased emission intensity upon EGF stimulation, mirroring the isoform‐specific response trends observed in FRK imaging (Figure 3E,F). Consistent with previous data, HRK‐A showed the highest FRET signal change, followed by HRK‐C and HRK‐B. Similarly, BRET spectral analysis demonstrated that all HRK variants produced emission peaks at 517 nm, with signal intensities increasing after EGF stimulation–most prominently in HRK‐A (Figure 3G,H). These findings validate the HRK platform as a dual‐mode biosensor capable of reliably capturing RAF‐KRAS interactions via both fluorescence‐ and luminescence‐based energy transfer. Beyond maintaining isoform‐specific response profiles and robust FRET performance, HRKs offer luminescence‐based detection suitable for low‐background or light‐sensitive applications, broadening their utility for diverse experimental contexts, including potential in vivo implementation.

### 2.4 Mutation‐Specific Detection of RAF‐KRAS Interactions using HRKs

**FIGURE 4:**
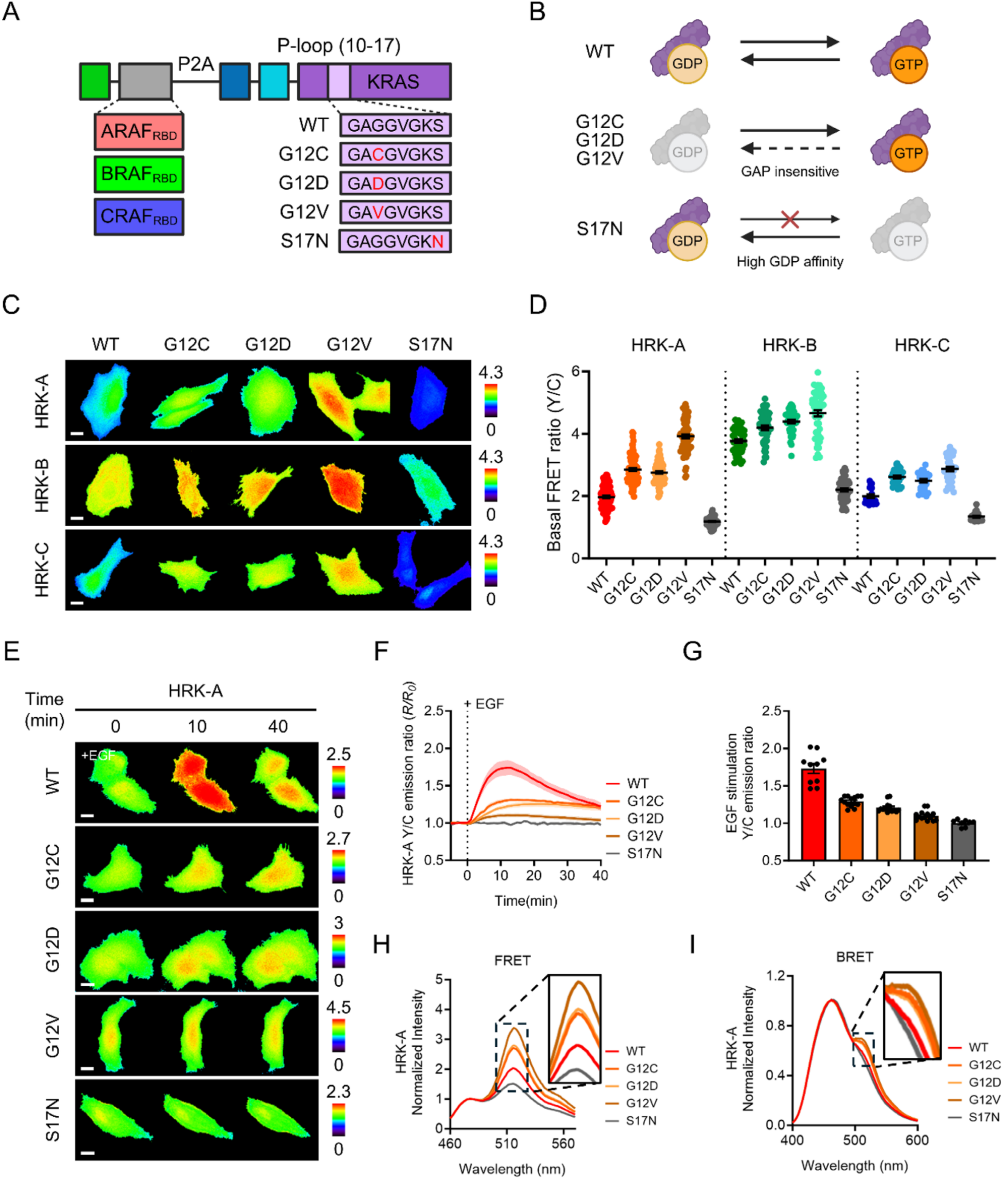
HRKs distinguish mutation‐specific RAF‐KRAS binding among KRAS variants. (A) Schematic representation of HRK biosensor variants. (B) Conceptual diagram illustrating GTP/GDP cycling properties and RAF‐binding capacities of wild‐type and mutant KRAS proteins. (C) Representative FRET/CFP ratio images of HeLa cells expressing HRK‐A, HRK‐B, or HRK‐C containing either wild‐type or mutant KRAS. Scale bar = 20 µm. (D) Quantification of basal FRET/CFP ratios corresponding to (C), shown as dot plots for each HRK construct (HRK‐A, n = 66–96; HRK‐B, n = 44–51; HRK‐C, n = 19–33). (E) Representative time‐lapse FRET/CFP ratio images of HeLa cells expressing HRK‐A variants following stimulation with 50 ng/mL EGF. Scale bar = 20 µm. (F) Time‐course of the mean normalized FRET/CFP ratio changes of HRK‐A variants before and after treatment with 50 ng/mL EGF (HRK‐A‐WT, n = 10; HRK‐A‐G12C, n = 14; HRK‐A‐G12D, n = 14; HRK‐A‐G12V, n = 14; HRK‐A‐S17N, n = 8). (G) Quantification of the peak FRET/CFP ratio derived from individual traces in (F), representing the maximal response of each HRK‐A variant to EGF stimulation. Each dot indicates a single cell. (H) Normalized FRET emission spectra of HRK‐A variants, aligned to the emission peak of donor fluorescent protein (n = 16 spectra). (I) Normalized BRET emission spectra of HRK‐A variants, aligned to the emission peak of NanoLuc luciferase (n = 3 spectra). Data are presented as mean ± SEM.

KRAS mutations, particularly those at codon 12 within the P‐loop domain, are well‐established drivers of oncogenic signaling by impairing intrinsic GTPase activity and locking KRAS in a constitutively active, GTP‐bound state [38, 39]. To investigate how these mutations influence RAF isoform‐specific binding dynamics in live cells, we generated HRK variants carrying the G12C, G12D, or G12V mutations, as well as the S17N variant that mimics the inactive GDP‐bound conformation (Figure 4A) [40, 41]. The oncogenic variants remain predominantly GTP‐bound due to impaired responsiveness to GAPs, whereas S17N remains inactive because of its high GDP‐binding affinity (Figure 4B).

Live‐cell baseline FRET/CFP imaging revealed that all three oncogenic mutants exhibited significantly higher basal FRET ratios than wild‐type KRAS across HRK‐A, HRK‐B, and HRK‐C (Figure 4C,D). This consistent elevation suggests strong, constitutive RAF‐KRAS complex formation in the mutant state, even in the absence of upstream stimulation. Among the mutants, G12V produced the highest basal signal, consistent with its severely impaired GTP hydrolysis and prolonged effector engagement [42]. In contrast, the S17N variant exhibited low FRET ratios across all constructs, validating its inactive conformation and inability to bind RAF. These results collectively indicate that oncogenic KRAS mutants maintain a preactivated state that stabilizes RAF binding across ARAF, BRAF, and CRAF. Upon stimulation with 50 ng/mL EGF, mutant constructs showed markedly attenuated dynamic responses compared to wild‐type (Figure 4E–G). While wild‐type HRKs displayed a characteristic transient rise in FRET/CFP ratio followed by a gradual decline–reflecting GEF‐mediated activation and GAP‐induced inactivation–KRAS mutant biosensors exhibited minimal or no increase in FRET ratio. This observation implies that RAF binding is already saturated in the basal state for these mutants. Moreover, the typical post‐peak decrease seen in wild‐type sensors was entirely absent in the mutants, consistent with their resistance to GAP‐mediated GTP hydrolysis and dissociation. These patterns support the interpretation that oncogenic KRAS variants exhibit constitutive activation, rendering them largely unresponsive to upstream signaling cues and resistant to downstream regulatory feedback.

To determine whether these mutation‐specific behaviors are conserved across RAF isoforms, we analyzed HRK‐B and HRK‐C variants in parallel (Figure S6). Both sensors exhibited the same mutation‐dependent trends observed in HRK‐A: elevated basal FRET in mutants, a lack of EGF‐induced signal enhancement, and absence of post‐stimulation decay. Notably, HRK‐B consistently displayed higher basal FRET ratios–particularly in the G12V variant–compared to HRK‐A or HRK‐C, suggesting that BRAF may have a stronger constitutive affinity for activated KRAS. This isoform‐dependent variation may reflect differential regulatory functions among RAF family members in KRAS‐driven signaling. FRET and BRET spectral analyses further supported these findings (Figure 4H,I; Figure S6D,E,I,J), with G12V consistently showing the strongest donor to acceptor energy transfer across both detection modes. Conversely, S17N remained spectrally silent, confirming its inactive state. These results demonstrate that HRKs robustly resolve mutation‐specific RAF‐KRAS interactions in live cells.

As an additional biochemical validation that the observed HRK signal patterns arise from correctly processed sensor components, we analyzed a dual 6×His‐tagged HRK‐A variant by SDS‐PAGE following IMAC‐based enrichment. In this construct, 6×His tags were placed at the N‐termini of the two P2A‐generated products, enabling simultaneous enrichment of both components. Recombinant protein purification was performed by a commercial service provider (NKMAXBio, Korea), and eluted fractions were analyzed by Coomassie‐stained SDS‐PAGE (Figure S7). Two predominant bands were detected at approximately 38.4 and 69.7 kDa, in close agreement with the predicted molecular weights of the P2A‐cleaved HRK‐A products. These results confirm efficient P2A‐mediated processing and support that the observed HRK intensity patterns originate from properly generated, discrete sensor components.

To further corroborate that the mutation‐dependent HRK signatures observed in live cells are preserved in an orthogonal biochemical context, we performed spectral emission measurements using clarified cell lysates (Figure S8A). Lysates prepared from HRK‐expressing cells retained the same rank‐order behavior observed by live‐cell imaging, with oncogenic KRAS variants exhibiting elevated basal signals and attenuated EGF‐induced dynamics relative to wild‐type. Moreover, spectral measurements collected under basal and EGF‐stimulated conditions reproduced the same directional responses seen in intact cells (Figure S8B–D). These findings indicate that HRK readouts consistently capture mutation‐dependent RAF–KRAS interaction states across both imaging‐ and lysate‐based measurements.

Taken together, this work highlights the ability of HRKs to distinguish wild‐type and oncogenic KRAS based on both basal complex formation and dynamic responsiveness. Their capacity to resolve mutation‐specific, isoform‐dependent, and regulatory‐resistant signaling behaviors underscores their potential as powerful tools for live‐cell KRAS profiling and therapeutic screening.

### 2.5 Real‐Time Monitoring of Mutation‐Selective KRAS Inhibition Using HRKs

**FIGURE 5:**
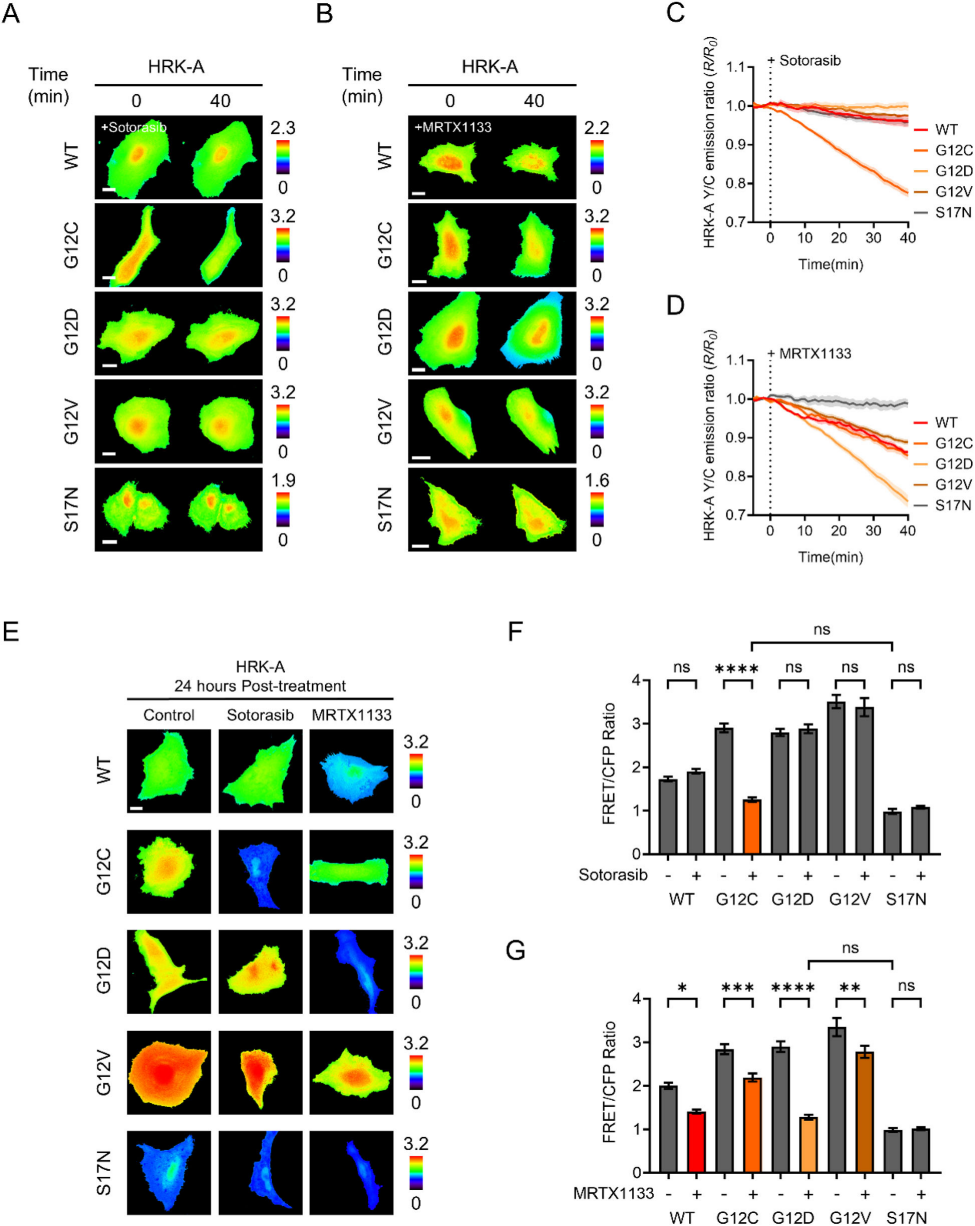
Evaluation of oncogenic KRAS inhibitor responses using real‐time FRET microscopy. (A) Representative time‐lapse FRET/CFP ratio images of HeLa cells expressing HRK‐A variants treated with 500 nM sotorasib. Scale bar = 20 µm. (B) Representative time‐lapse FRET/CFP ratio images of HeLa cells expressing HRK‐A variants treated with 100 nM MRTX1133. Scale bar = 20 µm. (C) Time‐course of the mean normalized FRET/CFP ratio following 500 nM sotorasib (n = 6–10) and (D) 100 nM MRTX1133 (n = 6–14). (E) Representative FRET/CFP ratio images of HeLa cells following 24 h treatment with 500 nM sotorasib, 100 nM MRTX1133, or 0.5 % (v/v) DMSO as a control. (F) Mean FRET/CFP ratios measured after 24 h treatment with 500 nM sotorasib (n = 13–55) and (G) 100 nM MRTX1133 (n = 13–58) across KRAS variants (^*^ *p* < 0.05, ^**^ *p* < 0.01, ^***^ *p* < 0.001, ^****^ *p* < 0.0001, ns: not significant). Data are presented as mean ± SEM, and statistical significance was calculated using two‐way ANOVA followed by Tukey's post hoc test.

To evaluate the ability of HRKs to monitor inhibition of oncogenic KRAS variants in a mutation‐specific manner, we assessed their response to two well‐characterized KRAS inhibitors: sotorasib (AMG‐510) [43], a covalent G12C‐specific inhibitor, and MRTX1133 [44], a potent non‐covalent compound selectively targeting G12D. Treatment with sotorasib led to a rapid and significant reduction in the FRET/CFP ratio exclusively in G12C‐expressing cells, indicating effective disruption of the RAF‐KRAS complex and validating the high specificity of this inhibitor (Figure 5A,C). No appreciable changes were detected in cells expressing G12D, G12V, or S17N, thereby confirming the selective covalent engagement of sotorasib with the cysteine residue at position 12 [45]. Unlike sotorasib, which forms a covalent bond, MRTX1133 binds non‐covalently and does not require the presence of a reactive cysteine [46]. Instead, it exhibits high affinity for the inactive, GDP‐bound conformation of KRAS, stabilizing this state and thereby blocking downstream signaling. In our experiments, MRTX1133 treatment led to the largest reduction in FRET signal in G12D‐expressing cells, consistent with strong inhibitor engagement (Figure 5B,D). Moderate reductions were also observed in wild‐type, G12C, and G12V variants, suggesting that MRTX1133 may still engage these KRAS forms to a limited extent–sufficient to partially disrupt preexisting RAF‐KRAS interactions, even if binding is less stable than in G12D [47, 48, 49]. The S17N variant showed no change in FRET ratio following MRTX1133 treatment. Since S17N mimics an inactive GDP‐bound form that does not bind RAF, the absence of signal reduction supports the interpretation that FRET decreases in other variants result from inhibitor‐induced dissociation of preformed RAF‐KRAS complexes. To further evaluate the extent of RAF‐KRAS inhibition beyond real‐time response, we performed endpoint imaging following 24 h pretreatment with KRAS inhibitors. In cells expressing the G12C sensor, sotorasib treatment reduced the FRET ratio to the level observed in the S17N sensor, indicating near‐complete disruption of RAF‐KRAS binding (Figure 5E, middle; Figure 5F). No appreciable signal change was observed in other KRAS variants, confirming the specificity of sotorasib for the G12C mutant. In contrast, pretreatment with MRTX1133 led to a global reduction in FRET ratio across all sensors except S17N, with the most pronounced inhibition observed in the G12D variant (Figure 5E, right; Figure 5G). Comparison with the S17N baseline revealed that MRTX1133 induced nearly complete inhibition in the G12D mutant, while only partial suppression was observed in the G12C and G12V variants.

### 2.6 Dose‐Response Analysis of KRAS Inhibitor Selectivity across KRAS Variants

**FIGURE 6:**
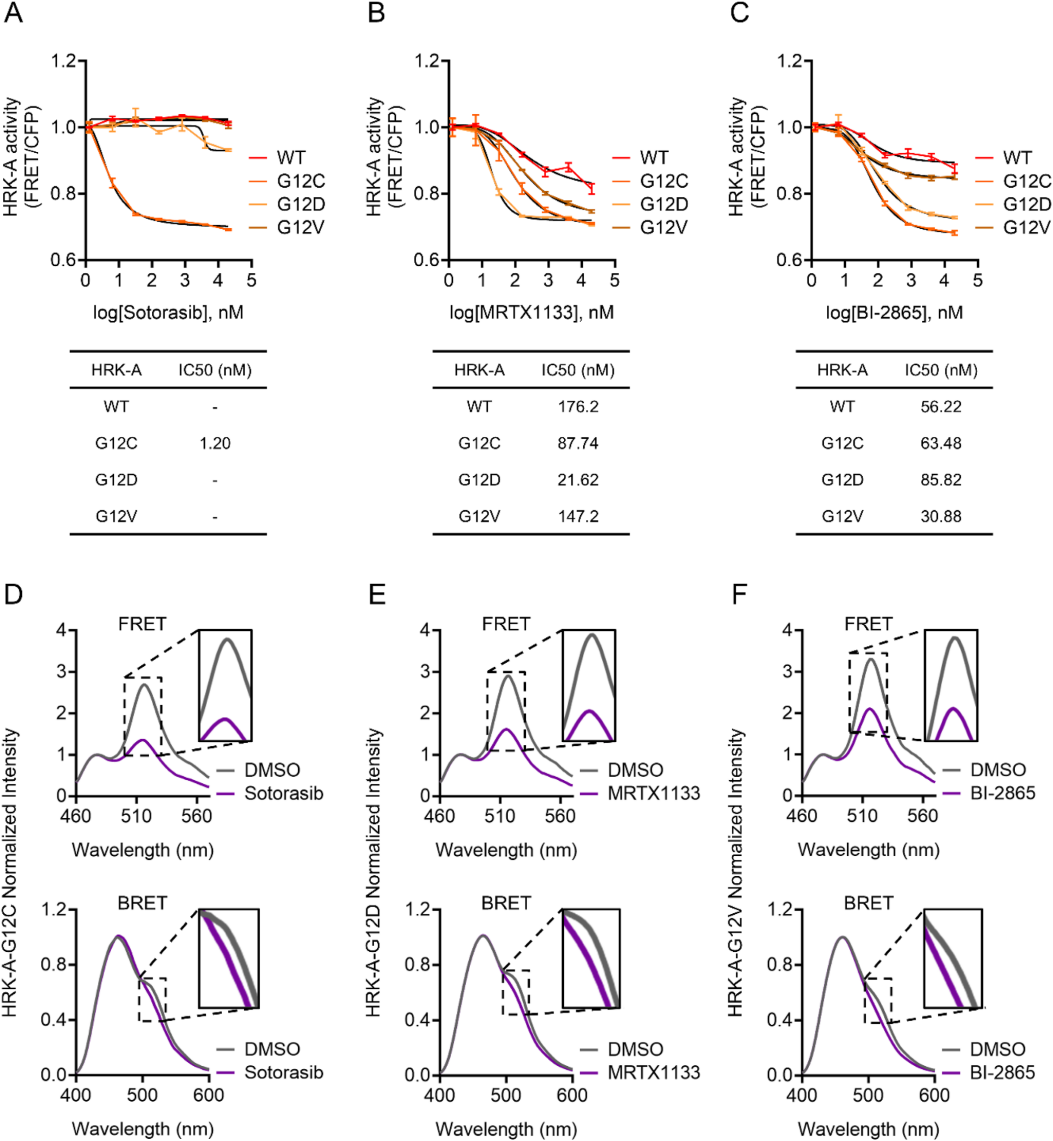
Differential KRAS variant inhibition revealed by FRET‐BRET microplate assays. (A) Dose‐response analysis of sotorasib (n = 4), (B) MRTX1133 (n = 4), and (C) BI‐2865 (n = 4) in HRK‐A‐expressing cells. IC_50_ values were calculated using nonlinear regression analysis. (D) Normalized FRET and BRET emission spectra of HRK‐A‐G12C in Lenti‐X 293T cells treated with 500 nM sotorasib or DMSO (0.5 % v/v, control) for 24 h (FRET, n = 3; BRET, n = 7). (E) Normalized FRET and BRET emission spectra of HRK‐A‐G12D in Lenti‐X 293T cells treated with 100 nM MRTX1133 or DMSO (0.5 % v/v, control) for 24 h (FRET, n = 4; BRET, n = 6). (F) Normalized FRET and BRET emission spectra of HRK‐A‐G12V in Lenti‐X 293T cells treated with 100 nM BI‐2865 or DMSO (0.5 % v/v, control) for 24 h (FRET, n = 3; BRET, n = 3). Data are presented as mean ± SEM.

To evaluate the inhibitory potency of KRAS‐targeted compounds across different KRAS variants, we performed a microplate‐based FRET assay using HRK‐A sensors treated with increasing doses of sotorasib, MRTX1133, or BI‐2865 (Figure 6A–C). Sotorasib showed selective inhibition of G12C with strong potency (IC_50_ = 1.20 nM), while no measurable response was observed in wild‐type, G12D, or G12V, consistent with its allele‐specific covalent mechanism. MRTX1133 exhibited the greatest effect in G12D (IC_50_ = 21.62 nM), but also reduced FRET signals in wild‐type (IC_50_ = 176.2 nM), G12C (IC_50_ = 87.74 nM), and G12V (IC_50_ = 147.2 nM), reflecting its broader inhibitory activity. BI‐2865, a pan‐KRAS inhibitor, decreased RAF‐KRAS binding across all variants, with comparable IC_50_ values in wild‐type (IC_50_ = 56.22 nM), G12C (IC_50_ = 63.48 nM), G12D (IC_50_ = 85.82 nM), and G12V (IC_50_ = 30.88 nM). Consistent with these live‐cell measurements, an additional in vitro dose‐response analysis produced broadly similar inhibitor‐response patterns for HRK‐A, providing an independent validation of variant‐dependent potency profiles across assay formats (Figure S9A–C). To further substantiate these observations, spectral analyses were performed to directly assess energy transfer changes. Sotorasib induced a clear loss of both FRET and BRET signals exclusively in G12C‐expressing cells (Figure 6D; Figures S10A and S11A), confirming its strict mutation specificity. MRTX1133 treatment resulted in reduced emission intensities across all variants, with the strongest suppression observed in G12D, followed by G12C, G12V, and wild‐type (Figure 6E; Figures S10B and S11B). BI‐2865 elicited uniform decreases in both FRET and BRET signals across all KRAS variants (Figure 6F; Figures S10C and S11C). Together, these results demonstrate that HRK biosensors not only detect mutation‐specific inhibition with high sensitivity, but also resolve variant‐dependent differences in inhibitory efficacy, enabling fine discrimination of inhibitor selectivity across oncogenic KRAS mutations.

## 3 Discussion

In this study, we report the development and validation of a set of genetically encoded biosensors, termed FRKs and HRKs, that enable real‐time, isoform‐specific, and mutation‐sensitive monitoring of RAF‐KRAS interactions in living cells. These biosensors integrate fluorescence‐ and luminescence‐based modalities, allowing dual detection through FRET and BRET, and offer a robust platform for dissecting KRAS signaling dynamics with high temporal resolution. KRAS mutations at codon 12 result in impaired GTP hydrolysis and constitutive activation of downstream pathways, such as RAF–MEK–ERK [38, 39]. While these oncogenic features are well established, live‐cell visualization of RAF‐KRAS binding dynamics has been limited by technical constraints. The biosensors presented here address these limitations by enabling real‐time monitoring of both association and dissociation events, with high temporal resolution, strong signal contrast, and isoform‐level specificity.

The FRK series allowed isoform‐specific dissection of RAF‐KRAS binding kinetics. Notably, FRK‐A (ARAF) exhibited the most pronounced EGF‐induced FRET response with the highest fold increase from baseline, whereas FRK‐B (BRAF) displayed the highest basal FRET ratio even in the absence of stimulation. This suggests that BRAF may have a higher basal affinity for KRAS or may more readily associate under resting conditions. Previous structural and biochemical studies have shown that BRAF exhibits strong membrane localization and constitutive kinase activity, which may contribute to elevated basal interactions with KRAS [50, 51]. In addition, even in studies utilizing only the RBD domains of RAF isoforms, BRAF‐RBD demonstrated the highest binding affinity to KRAS among the three isoforms [10, 28]. This indicates that the observed differences in basal signal may not solely stem from full‐length protein behavior but rather reflect intrinsic isoform‐specific affinity at the RBD level. In contrast, ARAF and CRAF often require upstream phosphorylation or additional regulatory inputs for effective activation and membrane engagement. Together, these findings suggest that the isoform‐dependent differences in basal FRET signals observed in FRKs are a consequence of both structural affinity at the RBD level and broader differences in regulatory activation thresholds.

To probe the reversibility and temporal regulation of RAF‐KRAS interactions, we employed targeted pharmacologic inhibitors. Gefitinib, an EGFR inhibitor, induced a rapid decrease in the FRET/CFP ratio across all FRK variants when applied at their respective peak timepoints, indicating effective blockade of upstream signaling and accelerated RAF‐KRAS dissociation. In contrast, MCP110 selectively disrupted CRAF‐KRAS interactions, consistent with its specificity for CRAF‐RBD. Notably, these dissociation events were clearly observable with our ratiometric biosensors. Previous studies using endpoint or intensiometric reporters often showed sustained RAF‐KRAS binding signals following EGF stimulation–even beyond the peak response–until exogenous inhibitors were applied [52, 53]. Such systems lacked the temporal sensitivity to capture signal attenuation, likely due to the limited resolution of GEFs and GAPs dynamics. In our system, by contrast, FRET ratio decline was observed following peak activation even without immediate pharmacologic intervention, reflecting endogenous GAP‐mediated GTP hydrolysis and RAF dissociation. This pattern underscores the ability of our sensors to report not only complex formation, but also regulated disassembly, thereby offering a more physiologically relevant and temporally resolved view of RAF‐KRAS signaling dynamics in living cells.

The development of HRKs involved a substantial structural modification, with NanoLuc luciferase inserted upstream of the ECFP fluorophore. Given the sensitivity of FRET to both intermolecular distance and dipole orientation between fluorophores, such an insertion posed a potential risk of altering energy transfer efficiency by disrupting spatial geometry. However, direct comparisons between HRK and FRK variants revealed no significant difference in basal or EGF‐stimulated FRET responses across RAF isoforms. This suggests that the modular configuration of the HRK design maintains an orientation and distance between donor and acceptor that remains permissive for efficient FRET. The preservation of signal fidelity despite architectural changes highlights the structural robustness and adaptability of the biosensor platform. Moreover, the added BRET functionality–enabled by substrate‐driven bioluminescence from Nluc–offers a low‐background, phototoxicity‐free alternative for applications in complex or light‐sensitive biological systems, including in vivo settings. Beyond validating the hybrid sensor design, HRKs also provided functional insights into mutation‐specific RAF engagement. All three oncogenic KRAS variants exhibited elevated basal FRET ratios relative to wild‐type, consistent with constitutive activation and enhanced effector binding. Notably, G12V, characterized by its severely impaired GTPase activity [42], consistently produced the highest FRET signal, suggesting that this mutation not only promotes activation but also prolongs RAF‐KRAS complex stability. These differences in basal association kinetics across mutants may influence downstream signaling and therapeutic response profiles. In contrast, the GDP‐preferring S17N mutant showed minimal basal binding and no response to EGF stimulation, reaffirming its utility as a negative control and further validating the selectivity of the biosensor platform.

Given the substantial biochemical differences between intact cells and clarified lysates, we initially anticipated that RAF‐KRAS interaction profiles might diverge across assay formats. However, with our optimized lysate assay conditions [54, 55], the mutation‐dependent HRK patterns closely recapitulated those observed in live cells, including elevated basal signals and attenuated EGF‐induced dynamics in oncogenic KRAS variants relative to wild‐type, while S17N remained low as a negative control. Moreover, lysate spectra acquired under basal and EGF‐stimulated conditions reproduced the same directional responses observed in intact cells. Together, these findings demonstrate that the HRKs report RAF‐KRAS interaction states robustly across assay formats, supporting their utility as a biochemical readout while retaining the key advantages of live‐cell, real‐time measurements for interpreting signaling regulation in a physiologically relevant context.

To further explore mutation‐selective inhibition using HRKs, we evaluated cellular responses to two mechanistically distinct KRAS inhibitors: sotorasib, a covalent G12C‐specific compound, and MRTX1133, a reversible inhibitor with reported selectivity for KRAS G12D. As expected, sotorasib induced a rapid and pronounced decrease in both FRET and BRET signals exclusively in cells expressing HRK‐A‐G12C, confirming its allele‐specific covalent binding and validating the sensitivity of the biosensor. In contrast, MRTX1133 treatment led to the largest signal reduction in G12D‐expressing cells but also induced moderate decreases in G12C and G12V variants. These responses, absent in the GDP‐locked S17N control, reflect genuine disruption of RAF‐KRAS interactions. Although originally designed as a G12D‐selective agent, our live‐cell biosensor data indicate that MRTX1133 exerts measurable inhibitory activity across multiple KRAS mutant backgrounds. This broader efficacy likely stems from its non‐covalent binding mode and capacity to engage the nucleotide‐binding pocket in conformationally flexible mutants. Unlike covalent inhibitors that require precise residue targeting, MRTX1133 can reversibly associate with GDP‐like conformations, potentially enabling it to partially inhibit other oncogenic KRAS variants under certain cellular conditions [46]. The HRK platform, by enabling real‐time quantification of binding dynamics and inhibitor response in live cells, reveals pharmacodynamic features that are not readily captured by endpoint or biochemical assays. These findings highlight the potential of MRTX1133 to function beyond its nominal target and demonstrate the value of HRKs in uncovering such extended inhibitory profiles that could inform pan‐KRAS therapeutic strategies.

In summary, we developed a dual‐mode biosensor platform capable of monitoring RAF‐KRAS interactions in live cells with isoform‐ and mutation‐specificity. By integrating FRET and BRET modalities, the HRKs enable flexible detection of both interaction formation and regulated dissociation, capturing signaling dynamics often missed in static or in vitro assays. The sensors revealed mutation‐dependent binding behaviors, GAP resistance, and selective responses to inhibitors such as sotorasib and MRTX1133. Notably, the HRK readouts were highly consistent between live‐cell and lysate‐based assays, indicating that the mutation‐dependent RAF‐KRAS interaction patterns are robust across experimental formats. Together, these biosensors provide a powerful toolset for dissecting RAS signaling in physiological contexts and for evaluating pharmacologic responses with high resolution.

## 4 Experimental Section

### 4.1 Cell Culture and Transfection

Lenti‐X 293T cells (Clontech, 632180; RRID: CVCL_4401) and HeLa cells (Korea Cell Line Bank, 10002; RRID: CVCL_0030) were cultured in Dulbecco's Modified Eagle Medium (DMEM; GenDEPOT, CM002‐050) supplemented with 10 % fetal bovine serum (FBS; Gibco, 16000–044) and 1 % penicillin‐streptomycin (100 U/mL penicillin and 100 µg/mL streptomycin; GenDEPOT, CA005). All cell lines were tested negative for mycoplasma contamination using CycleavePCR Mycoplasma Detection Kit (Takara, CY232). Cells were maintained at 37°C in a humidified incubator with 5 % CO_2_ and used for experiments at 70 %–80 % confluency. For live‐cell FRET imaging, HeLa cells were seeded in glass‐bottom confocal dishes (SPL, 200350). After 24 h of incubation, plasmid DNA was transfected using PEIpro reagent (Polyplus, 115‐010) according to the manufacturer's protocol. Prior to imaging, cells were washed with PBS (WELGENE, LB004‐02) and incubated in CO_2_‐independent medium (Gibco, 18045–088) supplemented with 0.5 % FBS, 1 % penicillin‐streptomycin, and 1× GlutaMAX (Gibco, 35050–061). For FRET and BRET spectral analysis, Lenti‐X 293T cells were transfected with FRK or HRK constructs in 6‐well plates (SPL, 30006). After 24 h, cells were harvested and resuspended in clear DMEM (GenDEPOT, CM004‐310) supplemented with 0.5 % FBS and 1 % penicillin‐streptomycin. Cell suspensions were then transferred to clear‐bottom 96‐well black plates (Greiner Bio‐One, 655087) or white‐bottom 96‐well plates (SPL, 30196) for spectral measurements.

### 4.2 Plasmid Construction

The FRET‐based RAF‐KRAS biosensor (FRK) was constructed through sequential PCR amplification, restriction enzyme digestion, and ligation, as schematically illustrated in Figure 1. The donor fluorophore ECFP was amplified from Eevee‐ROCK (a gift from the laboratory of Michiyuki Matsuda) and digested with NotI and BspEI. The acceptor fluorophore mNeonGreen was amplified from ER‐mNeonGreen (Addgene #137804) and digested with BamHI and XhoI. The CRAF‐RBD‐P2A linker and full‐length KRAS were amplified from G‐KRAS (provided by the laboratory of Won Do Heo) and digested with XhoI/NotI and NheI/PspOMI, respectively. After construction of FRK‐C, ARAF‐RBD, and BRAF‐RBD were amplified from pBABEpuro‐ARAF (Addgene #51076) and BRAF (Addgene #40775), respectively, and digested with XhoI and BglII to replace the CRAF‐RBD region. To generate the hybrid RAF‐KRAS biosensor (HRK), NanoLuc luciferase was amplified from pNCS‐Antares (Addgene #74279), digested with NotI and PspOMI, and inserted upstream of ECFP. HRK‐A was subcloned into pHEK293 Ultra Expression Vector I (Takara Bio, Cat. #3390) by BamHI/SalI restriction cloning to generate pHEK‐HRK‐A. To generate a dual 6×His‐tagged HRK‐A variant, annealed oligonucleotide duplexes (Macrogen, Korea) encoding a 6×His tag were inserted in‐frame immediately upstream of the mNeonGreen coding sequence and immediately downstream of the NanoLuc luciferase coding sequence, without an additional linker.

### 4.3 Chemical and Drug Compounds

Epidermal growth factor (EGF; Sigma–Aldrich, E9644) was reconstituted in 10 mM acetic acid. Gefitinib (MedChemExpress, HY‐50895), MCP110 (Selleck Chemical, S6905), sotorasib (MedChemExpress, HY‐114277), MRTX1133 (MedChemExpress, HY‐134813), and BI‐2865 (MedChemExpress, HY‐153724) were dissolved in dimethyl sulfoxide (DMSO; Biosesang, AC4002‐050‐00). DMSO was used as a vehicle control at a final concentration of 0.5 % (v/v).

### 4.4 Microscopy and Image Acquisition

Consistent with earlier descriptions, live‐cell FRET imaging was performed using HeLa cells seeded in glass‐bottom confocal dishes and transfected with FRK or HRK biosensor constructs. Imaging was conducted on a Leica DMi8 inverted fluorescence microscope equipped with an LED8 light source, an HC PL APO 100×/1.30 oil immersion objective, a differential interference contrast (DIC) module, a K5 sCMOS camera (Leica Microsystems), and a stage‐top incubator with temperature and CO_2_ control to maintain physiologic conditions throughout imaging. FRET excitation was provided at 440 nm, and emission was collected at 460 nm (ECFP) and 535 nm (mNeonGreen) using a CYR71010 filter cube (Leica, 11525416) mounted in a motorized emission filter wheel (Leica, EFW‐LED8). Image acquisition was performed using LAS X software (Leica), and camera settings such as exposure time and gain were standardized across all imaging sessions to ensure accurate and reproducible ratiometric quantification. To induce RAF‐KRAS binding, EGF was directly added to the imaging medium at a final concentration of 50 ng/mL. The FRET/CFP ratio was continuously monitored in real‐time following stimulation to determine the peak interaction timepoints for each biosensor construct. For upstream and isoform‐specific inhibition, cells were subsequently treated with 400 nM gefitinib or 100 nM MCP110 at peak FRET timepoints, as determined empirically for each construct. For mutation‐selective inhibition assays, cells expressing KRAS wild‐type or mutant biosensors were treated with 500 nM sotorasib or 100 nM MRTX1133. For compound experiments, DMSO was used as a vehicle control. FRET/CFP images were acquired every 1 min for a total duration of 40 min after compound addition to capture dynamic changes in RAF‐KRAS interaction. For endpoint inhibition imaging, cells were pretreated with 500 nM sotorasib or 100 nM MRTX1133 for 24 h prior to acquisition of FRET/CFP ratio images under the same microscope settings. To assess whether FRK expression perturbs downstream MAPK signaling, ERK activity was monitored in mock‐transfected vs. FRK‐A‐expressing cells using an ERK kinase translocation reporter (ERKKTR‐mRuby2) under basal and 50 ng/mL EGF‐stimulated conditions. All fluorescence imaging experiments were independently replicated at least three times under matched conditions to ensure reproducibility and robustness of observed signal changes.

### 4.5 FRET Ratio Analysis

For quantitative analysis of FRET signal intensity, the plasma membrane was selected as the region of interest (ROI) in cells expressing FRK or HRK biosensors. For each image, background fluorescence was measured in a region devoid of cells and subtracted from the ROI signal in both the FRET and ECFP channels to correct for nonspecific signals and camera noise. The background‐corrected FRET/CFP ratio was calculated on a pixel‐by‐pixel basis using the following equation:

$$FRET/CFP\;ratio=\frac{\left[I\left(FRET_{ROI}\right)-I\left(FRET_{bg}\right)\right]}{\left[I\left(ECFP_{ROI}\right)-I\left(ECFP_{bg}\right)\right]}$$

Here, *I* denotes the fluorescence intensity measured in the specified channel and region. The resulting FRET ratio images were visualized in intensity‐modified display mode, where the color of each pixel reflects the FRET/CFP intensity ratio.

### 4.6 Calculation of t_1/2_

To quantitatively assess RAF‐KRAS dissociation dynamics following treatment with gefitinib or MCP110, time‐lapse FRET/CFP ratio data were fitted to a single‐phase exponential decay model. In this analysis, time zero was defined as the point of maximal RAF‐KRAS interaction, corresponding to the peak FRET ratio. The subsequent decline in signal reflects inhibitor‐induced dissociation of the RAF‐KRAS complex and was modeled using the following equation:

$$R\left(x\right)=\left(R\left(p\right)-1\right)\cdot e^{-Kx}+1$$

In this equation, *R(x)* denotes the FRET/CFP ratio at time x, and *R(p)* (also referred to as *R(0)*) represents the FRET ratio at the peak of RAF‐KRAS binding. The parameter *K* corresponds to the rate constant of dissociation, and the plateau value was fixed at 1 to represent the basal (pre‐treatment) FRET ratio. The half‐life of RAF‐KRAS dissociation (t_1/2_) was calculated using the standard exponential decay relation:

$$t_{1/2}=\frac{\ln\;2}{K}$$

All curve fitting and kinetic parameter extraction were performed using GraphPad Prism version 10.1.2 (GraphPad Software).

### 4.7 FRET Spectral Analysis

After transfected cells were harvested and resuspended in clear DMEM, 1 × 10^6^ cells were seeded into clear‐bottom 96‐well black plates and incubated for 24 h prior to subsequent experiments. To assess RAF‐KRAS binding dynamics, EGF was added at a final concentration of 50 ng/mL, and fluorescence emission spectra were recorded 10 min post‐treatment. For evaluation of mutation‐selective inhibition, 500 nM sotorasib, 100 nM MRTX1133, and 100 nM BI‐2865 were applied in separate wells, and emission spectra were acquired 24 h after drug treatment. All drug treatments were performed in parallel wells, and DMSO was used as a vehicle control. For all experiments, fluorescence emission was measured from 460 to 570 nm following excitation at 434 ± 10 nm. To correct for background autofluorescence and normalize signal intensity, mean fluorescence values from untransfected control cells were subtracted from those of transfected cells at each emission wavelength. The resulting background‐corrected signal was then normalized using the following equation:

$$Normalized\;Intensity=\frac{\left(Ix-Ux\right)}{\left(I_{477}-U_{477}\right)}$$

Where *Ix* and *Ux* represent the fluorescence intensities at wavelength x for transfected and untransfected samples, respectively. The signal at 477 nm, corresponding to the ECFP donor emission, was used as a normalization reference to assess relative FRET intensity changes at 517 nm.

### 4.8 BRET Spectral Analysis

BRET spectral measurements were performed using the same experimental setup as described for FRET spectral analysis, except that cells were seeded into white‐bottom 96‐well plates instead of clear‐bottom black plates. After 24 h of incubation, cells were treated under the same conditions and for the same durations as in the FRET experiments. NanoBRET Nano‐Glo Substrate (Promega, N1662) was added to each well at a final volume of 0.2 µL per well. In all cases, the substrate was added 5 min prior to spectral acquisition to align signal stabilization. For EGF‐stimulated samples, luminescence spectra were recorded 10 min after EGF treatment. For inhibitor‐treated samples, luminescence spectra were acquired 24 h after drug treatment. Luminescence spectra were collected over a range of 400–600 nm and normalized to the donor emission peak at 460 nm, corresponding to the maximum emission of NanoLuc luciferase. All FRET and BRET spectral measurements were performed using the CLARIOstar Plus microplate reader (BMG Labtech), with identical detection settings applied across both modes.

### 4.9 FRET Ratio‐Based IC_50_ Measurement

Lenti‐X 293T cells transfected with HRK‐A variants were seeded into clear‐bottom 96‐well black plates and treated with 5 fold serial dilutions ranging from 1 nM to 20 µM of sotorasib, MRTX1133, or BI‐2865. After 24 h incubation, fluorescence intensities were measured using excitation at 434 ± 10 nm, and emission was detected at 477 nm (CFP) and 517 nm (FRET). The FRET ratio was calculated by dividing the FRET intensity by the CFP intensity. IC_50_ values were determined by nonlinear regression analysis (log[inhibitor] vs. response with variable slope) using GraphPad Prism version 10.1.2.

### 4.10 Cell Lysis

Cells at 70 %–80 % confluency were transfected with HRK constructs in T25 flasks (SPL, 70025) and incubated for 24 h. After removing the medium, cells were rinsed once with cold PBS and lysed using 500 µL of IP lysis buffer (Thermo Scientific, 87788) supplemented with Halt Protease Inhibitor Cocktail (Thermo Scientific, 78442) and 10 mM MgCl_2_ on ice. The lysates were collected and centrifuged at 13 000 rpm for 15 min at 4°C to remove debris. Supernatants were transferred to clear‐bottom 96‐well black plates and incubated at 4°C for another 20 min before measurement. For in vitro dose‐response (IC_50_) analysis, lysates were incubated with serial dilutions of sotorasib, MRTX1133, or BI‐2865 (with matched DMSO as the vehicle control) prior to plate‐based FRET measurement. IC50 values were determined by nonlinear regression of the dose‐response curves. Spectral acquisition was performed as described in the FRET spectral analysis section.

### 4.11 Statistical Analysis

All data are presented as mean ± standard error of the mean (SEM). Statistical analyses were performed using GraphPad Prism version 10.1.2. Differences between the two groups were evaluated using unpaired, two‐tailed Student's *t*‐tests. For comparison of three or more groups, one‐way or two‐way analysis of variance (ANOVA) was used as appropriate, followed by Tukey's multiple comparison test.

## Author Contributions

Jeong‐Min Go contributed to investigation, experiments, formal analysis, data curation, methodology, and wrote the initial draft. Daehee Lee, Minji Kim, Kiseok Han, Gyuho Choi, Chanhui Song, Sanghyun Ahn, Yerim Lee, and Jinyoung Lee contributed to investigation, experiments, formal analysis, data curation, and methodology. Yingxiao Wang contributed to data interpretation and revised the manuscript. Jung‐Soo Suh, Hwayoung Yun, and Tae‐Jin Kim conceptualized and supervised the project, acquired funding, and wrote and revised the manuscript.

## Conflicts of Interest

The authors declare no conflicts of interest.

## Supporting information




**Supporting File**: advs74415‐sup‐0001‐SuppMat.docx.

## Data Availability

The data that support the findings of this study are available in the supplementary material of this article.
