# Supplementary material for: Real‐Time Visualization of Isoform‐Specific RAF‐KRAS Interactions in Living Cells Using FRET‐BRET Hybrid Biosensors
Source: Adv Sci (Weinh). 2026 Feb 12;13(23):e15654. doi: 10.1002/advs.202515654 (PMC13104123; doi:10.1002/advs.202515654)
Supplement: Supplementary file 1 — Supporting File: advs74415‐sup‐0001‐SuppMat.docx. [file ADVS-13-e15654-s001.docx]

Supporting Information

**Real-Time Visualization of Isoform-Specific RAF-KRAS Interactions in Living Cells Using FRET-BRET Hybrid Biosensors**

Jeong-Min Go, Dahee Lee, Minji Kim, Kiseok Han, Gyuho Choi, ChanHui Song, Sanghyun Ahn, Yerim Lee, Jinyoung Lee, Yingxiao Wang, Jung-Soo Suh*, Hwayoung Yun*, and Tae-Jin Kim*


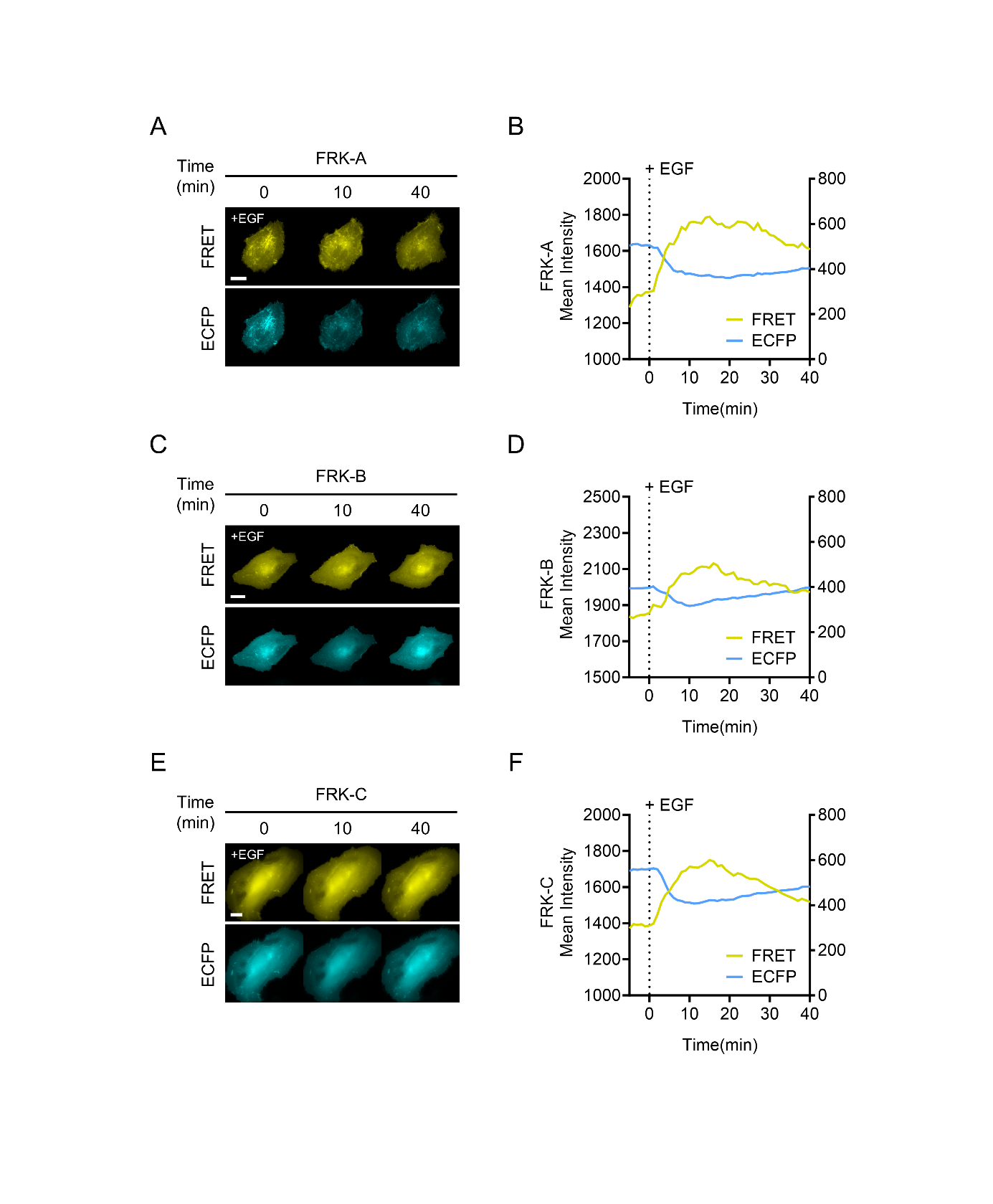


**Figure S1. Validation of FRK performance by direct measurement of FRET and ECFP intensities.** (A) Representative fluorescence images of FRET (yellow) and ECFP (cyan) channels in HeLa cells expressing FRK-A, acquired before and after stimulation with 50 ng/mL EGF. (B) Time-course of the mean FRET and ECFP channel intensities in HeLa cells expressing FRK-A (n = 8). Scale bar = 20 μm. (C) Representative fluorescence images of FRET and ECFP channels in HeLa cells expressing FRK-B, acquired before and after stimulation with 50 ng/mL EGF. (D) Time-course of the mean FRET and ECFP channel intensities in HeLa cells expressing FRK-B (n = 4). Scale bar = 20 μm. (E) Representative fluorescence images of FRET and ECFP channels in HeLa cells expressing FRK-C, acquired before and after stimulation with 50 ng/mL EGF. (F) Time-course of the mean FRET and ECFP channel intensities in HeLa cells expressing FRK-C (n = 6). Scale bar = 20 μm. Images in (A), (C), and (E) were taken from the same time-lapse datasets used to generate the FRET/CFP ratio images in Figure 1.


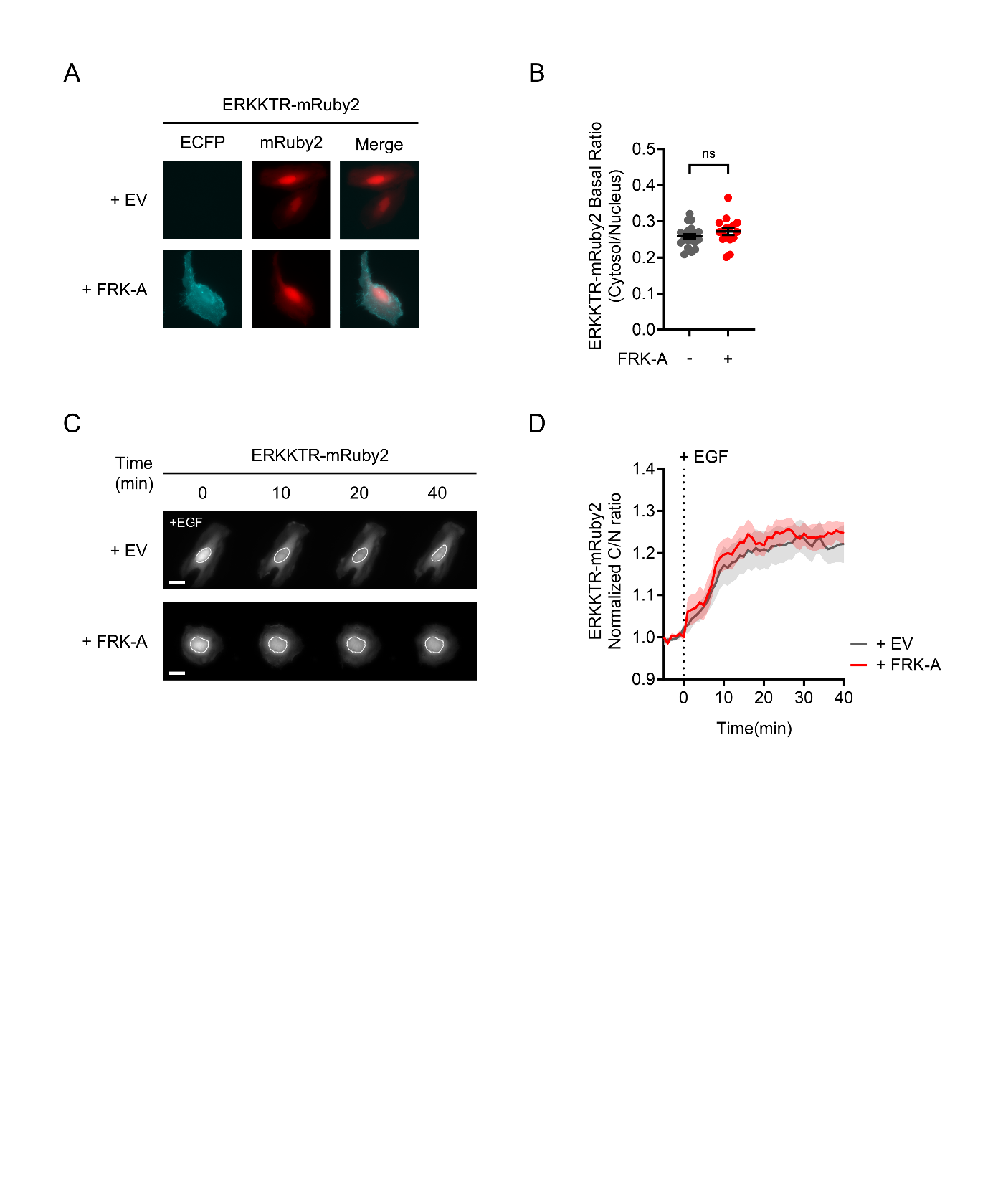


**Figure S2. Comparable ERK KTR responses in control and FRK-A-expressing cells.** (A) Representative fluorescence images of HeLa cells expressing ERKKTR-mRuby2 together with empty vector (EV) or FRK-A. ECFP channel indicates FRK-A expression, mRuby2 reports ERK KTR localization, and merged images are shown. Scale bar = 20 μm. (B) Quantification of basal ERK activity measured as the cytosol-to-nucleus (C/N) ratio of ERKKTR-mRuby2 in EV- or FRK-A-expressing cells (n = 16-21; ns: not significant). (C) Representative time-lapse images of ERKKTR-mRuby2 following stimulation with 50 ng/mL EGF in EV- or FRK-A-expressing cells. Scale bar = 20 μm. Nuclear regions used for quantification are outlined. (D) Time-course of normalized ERKKTR-mRuby2 C/N ratio following EGF stimulation (n = 10-11).


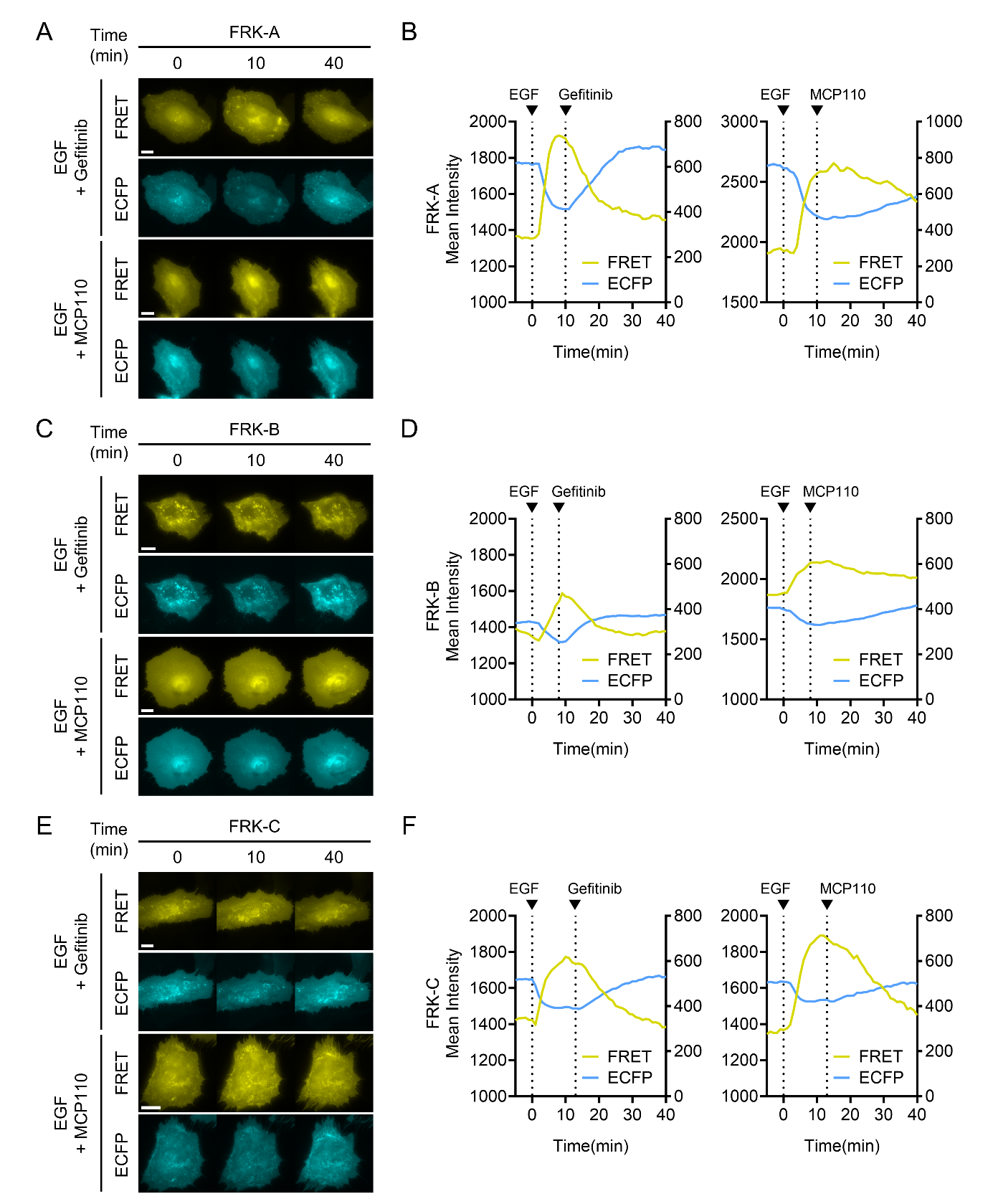


**Figure S3. Validation of inhibitor-induced FRK responses by direct measurement of FRET and ECFP intensities.** (A) Representative fluorescence images of FRET and ECFP channels in HeLa cells expressing FRK-A following stimulation with 50 ng/mL EGF and subsequent treatment with gefitinib or MCP110 at the indicated time points. Scale bar = 20 μm. (B) Time-course of the mean FRET and ECFP channel intensities in HeLa cells expressing FRK-A during EGF stimulation and inhibitor treatment (n = 5). (C) Representative fluorescence images of FRET and ECFP channels in HeLa cells expressing FRK-B under the indicated conditions. Scale bar = 20 μm. (D) Time-course of the mean FRET and ECFP channel intensities in HeLa cells expressing FRK-B under the indicated conditions (n = 5). (E) Representative fluorescence images of FRET and ECFP channels in HeLa cells expressing FRK-C under the indicated conditions. Scale bar = 20 μm. (F) Time-course of the mean FRET and ECFP channel intensities in HeLa cells expressing FRK-C under the indicated conditions (n = 6). Vertical dashed lines indicate the time points of EGF stimulation and inhibitor addition. Images in (A), (C), and (E) were taken from the same time-lapse datasets used to generate the FRET/CFP ratio images in Figure 2.


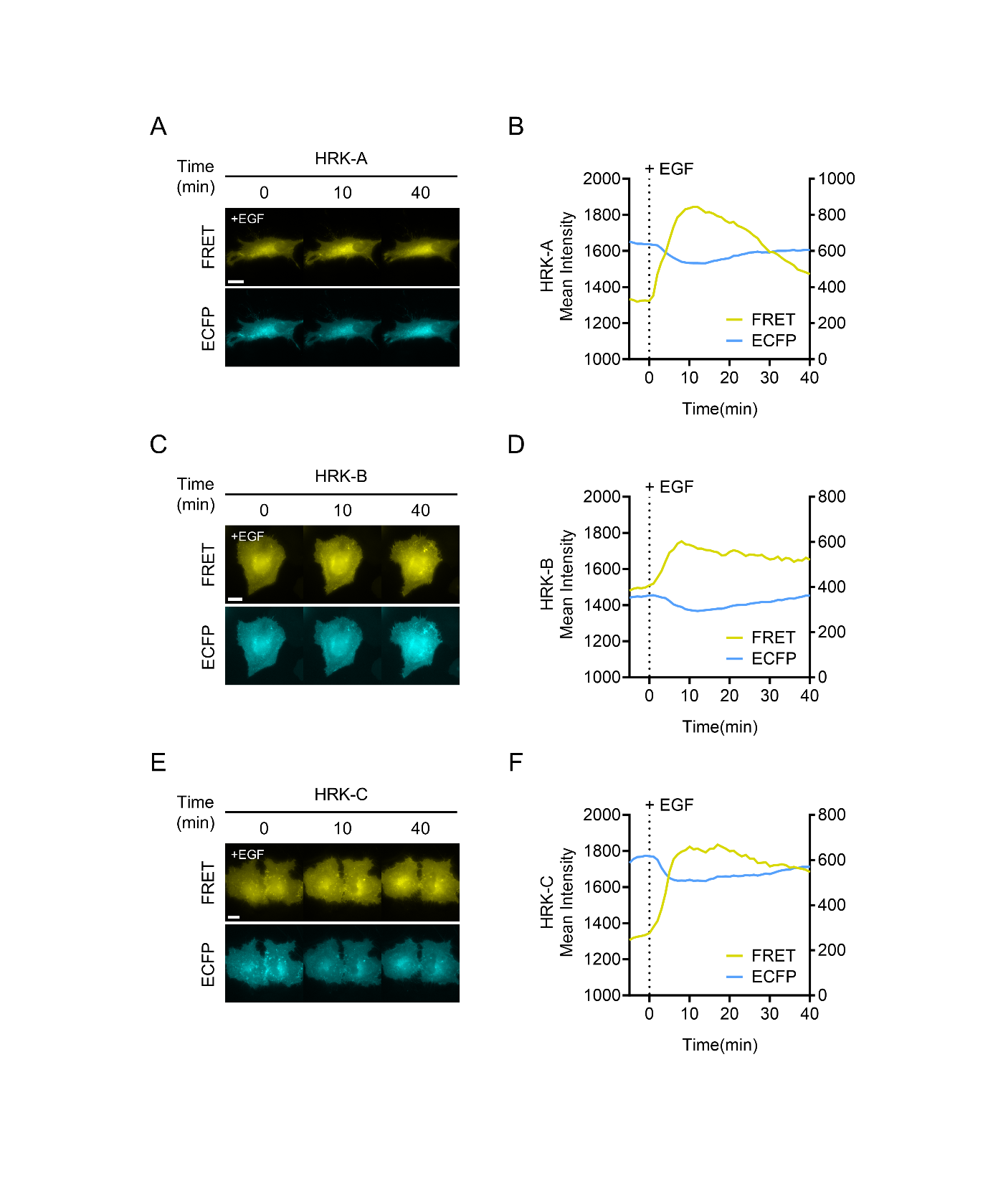


**Figure S4. Validation of HRK performance by direct measurement of FRET and ECFP intensities.** (A) Representative fluorescence images of FRET and ECFP channels in HeLa cells expressing HRK-A, acquired before and after stimulation with 50 ng/mL EGF. (B) Time-course of the mean FRET and ECFP channel intensities in HeLa cells expressing HRK-A (n = 6). Scale bar = 20 μm. (C) Representative fluorescence images of FRET and ECFP channels in HeLa cells expressing HRK-B, acquired before and after stimulation with 50 ng/mL EGF. (D) Time-course of the mean FRET and ECFP channel intensities in HeLa cells expressing HRK-B (n = 6). Scale bar = 20 μm. (E) Representative fluorescence images of FRET and ECFP channels in HeLa cells expressing HRK-C, acquired before and after stimulation with 50 ng/mL EGF. (F) Time-course of the mean FRET and ECFP channel intensities in HeLa cells expressing HRK-C (n = 6). Scale bar = 20 μm. Images in (A), (C), and (E) were taken from the same time-lapse datasets used to generate the FRET/CFP ratio images in Figure 3.


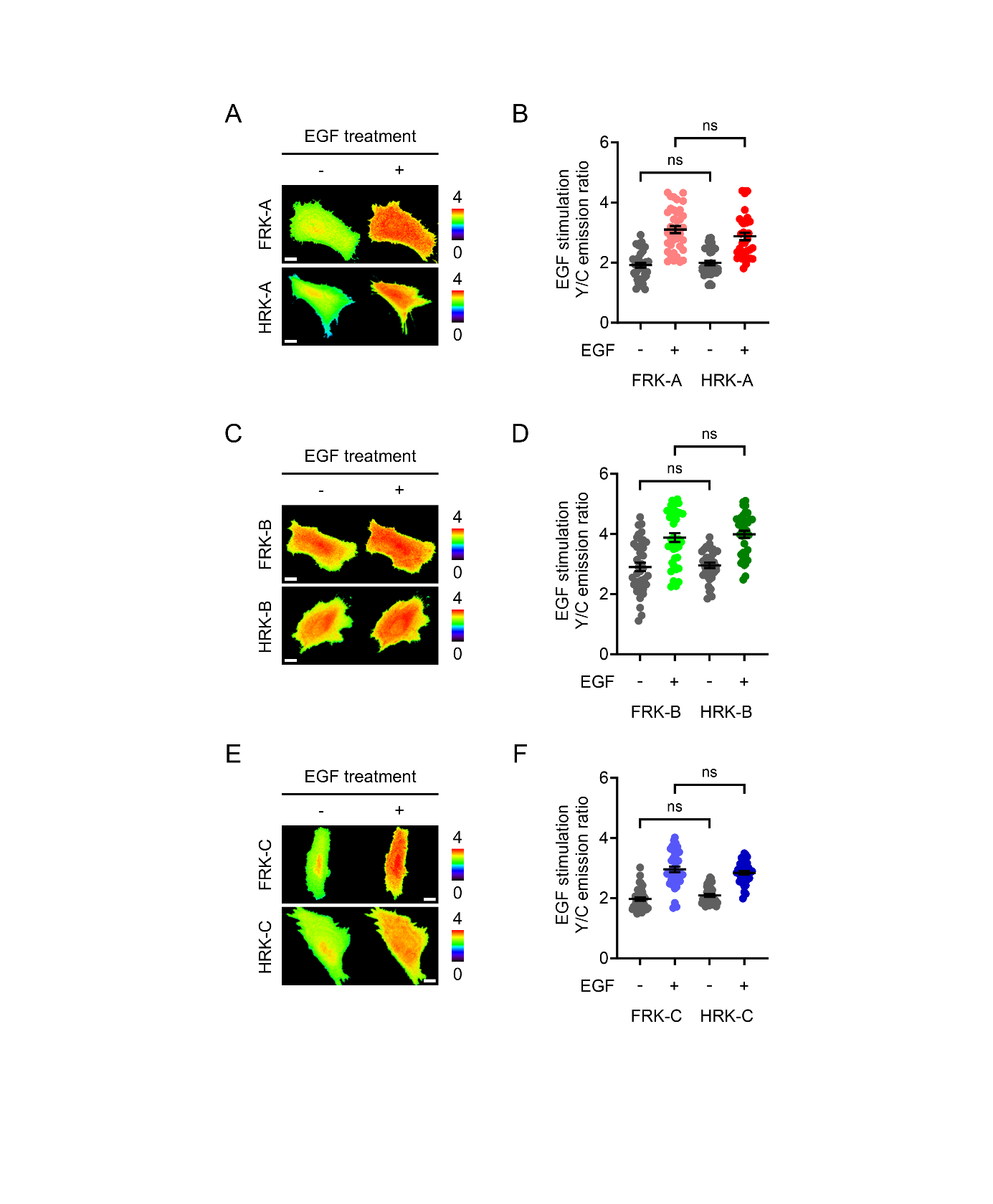


**Figure S5. Comparison of FRK and HRK biosensors to assess structural impact of Nluc insertion.** (A) Representative FRET/CFP ratio images and (B) corresponding quantification of HeLa cells expressing FRK-A or HRK-A before and 10 minutes after 50 ng/mL EGF stimulation (n = 35-37, ns: not significant). Scale bar = 20 μm. (C) Representative FRET/CFP ratio images and (D) corresponding quantification of HeLa cells expressing FRK-B or HRK-B before and 10 minutes after 50 ng/mL EGF stimulation (n = 36-39, ns: not significant). Scale bar = 20 μm. (E) Representative FRET/CFP ratio images and (F) corresponding quantification of HeLa cells expressing FRK-C or HRK-C before and 10 minutes after 50 ng/mL EGF stimulation (n = 38-40, ns: not significant). Scale bar = 20 μm. Data are shown as mean ± SEM. Statistical significance was determined using two-way ANOVA followed by Tukey’s post hoc test.


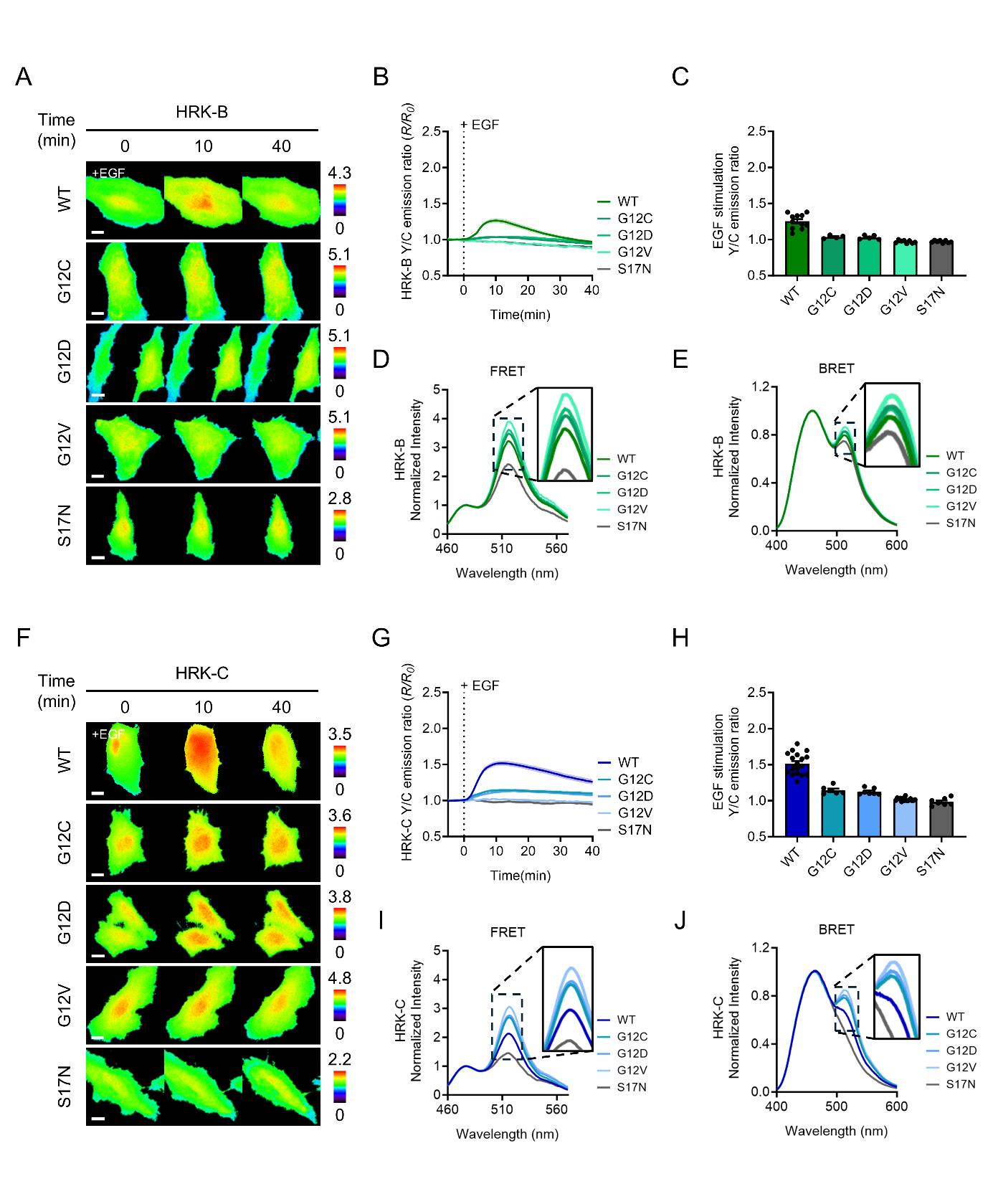


**Figure S6. Comparison of HRK-B and HRK-C variants.** (A) Representative time-lapse FRET/CFP ratio images of HeLa cells expressing HRK-B variants following stimulation with 50 ng/mL EGF. Scale bar = 20 μm. (B) Time-course of the mean normalized FRET/CFP ratio changes of HRK-B variants before and after treatment with 50 ng/mL EGF (HRK-B-WT, n = 11; HRK-B-G12C, n = 4; HRK-B-G12D, n = 5; HRK-B-G12V, n = 8; HRK-B-S17N, n = 8). The HRK-B-WT trace in (B) is reproduced from Figure 3C for comparison. (C) Quantification of the peak FRET/CFP ratio derived from individual traces in (B), representing the maximal response of each HRK-B variant to EGF stimulation. Each dot indicates a single cell. (D) Normalized FRET emission spectra of HRK-B variants, aligned to the emission peak of donor fluorescent protein (n = 17-18). (E) Normalized BRET emission spectra of HRK-B variants, aligned to the emission peak of NanoLuc luciferase (n = 9). Data are presented as mean ± SEM. (F) Representative time-lapse FRET/CFP ratio images of HeLa cells expressing HRK-C variants following stimulation with 50 ng/mL EGF. Scale bar = 20 μm. (G) Time-course of the mean normalized FRET/CFP ratio changes of HRK-C variants before and after treatment with 50 ng/mL EGF (HRK-C-WT, n = 18; HRK-C-G12C, n = 6; HRK-C-G12D, n = 7; HRK-C-G12V, n = 15; HRK-C-S17N, n = 7). The HRK-C-WT trace in (G) is reproduced from Figure 3C for comparison. (H) Quantification of the peak FRET/CFP ratio derived from individual traces in (G), representing the maximal response of each HRK-C variant to EGF stimulation. Each dot indicates a single cell. (I) Normalized FRET emission spectra of HRK-C variants, aligned to the emission peak of donor fluorescent protein (n = 11). (J) Normalized BRET emission spectra of HRK-C variants, aligned to the emission peak of NanoLuc luciferase (n = 6).


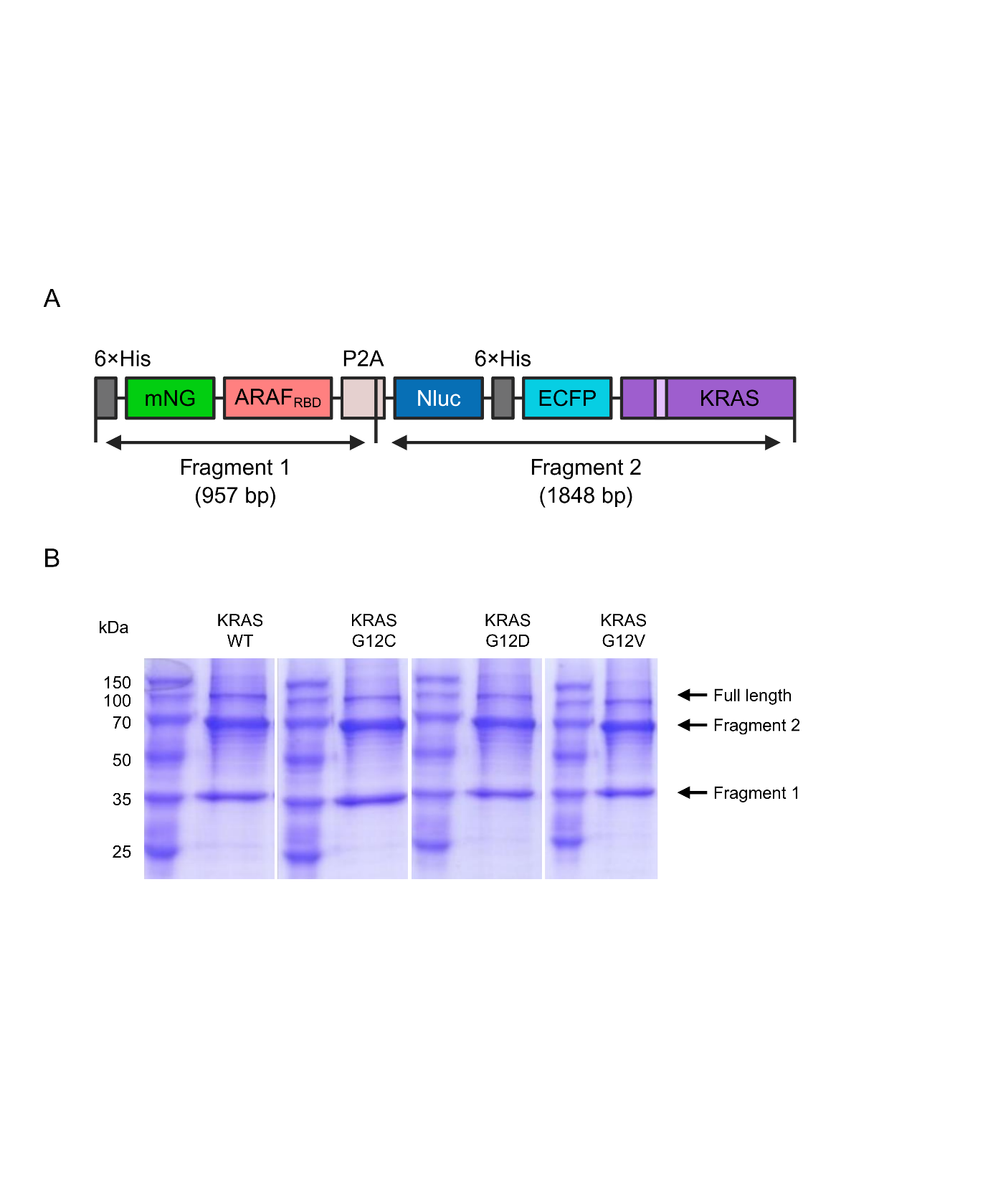


**Figure S7. Validation of P2A cleavage using dual 6×His-tagged HRK-A constructs.** (A) Schematic design of dual 6×His-tagged HRK-A constructs in which both P2A-derived products carry an N-terminal 6×His-tag (Fragment 1: mNeonGreen-ARAF-RBD; Fragment 2: NanoLuc-ECFP-KRAS), enabling simultaneous enrichment and detection of cleavage products. (B) Coomassie-stained SDS-PAGE of IMAC-enriched proteins from cells expressing the indicated KRAS variants, showing both P2A-cleaved fragments together with a minor full-length band corresponding to uncleaved HRK-A. Molecular weight markers (kDa) are shown on the left.


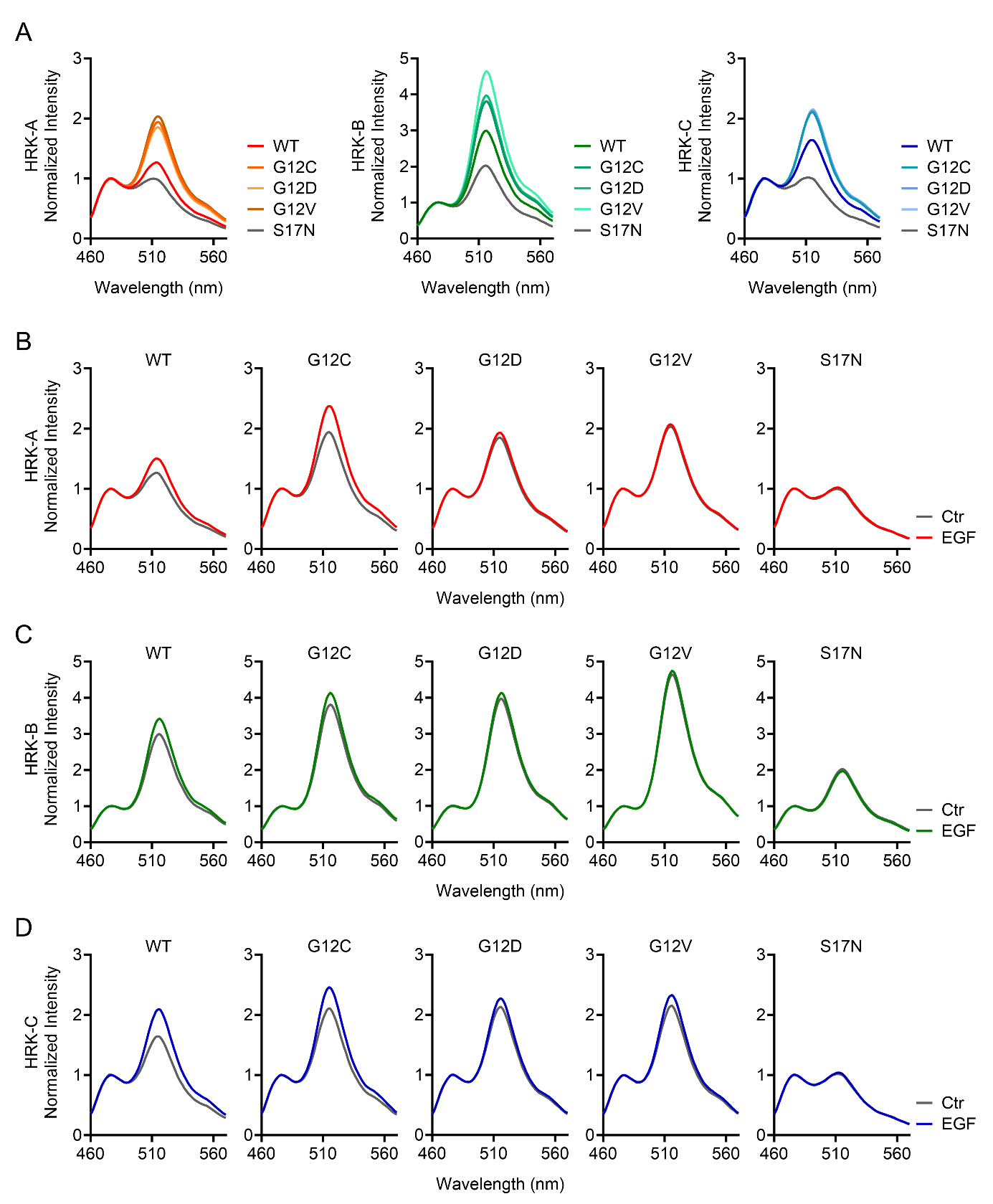


**Figure S8. Spectral FRET profiles of HRK variants across KRAS mutants under basal and EGF-stimulated conditions.** (A) Normalized FRET emission spectra of HRK-A, HRK-B, and HRK-C variants harboring the indicated KRAS mutations (WT, G12C, G12D, G12V, and S17N) under basal conditions. Spectra were normalized to enable comparison of relative emission profiles across variants. (B) Normalized FRET emission spectra of HRK-A variants measured under control conditions and 10 minutes after stimulation with 50 ng/mL EGF. (C) Normalized FRET emission spectra of HRK-B variants measured under control conditions and 10 minutes after stimulation with 50 ng/mL EGF. (D) Normalized FRET emission spectra of HRK-C variants measured under control conditions and 10 minutes after stimulation with 50 ng/mL EGF. All spectral measurements were performed in vitro in lysis buffer supplemented with 10 mM MgCl_2_ and protease inhibitors.


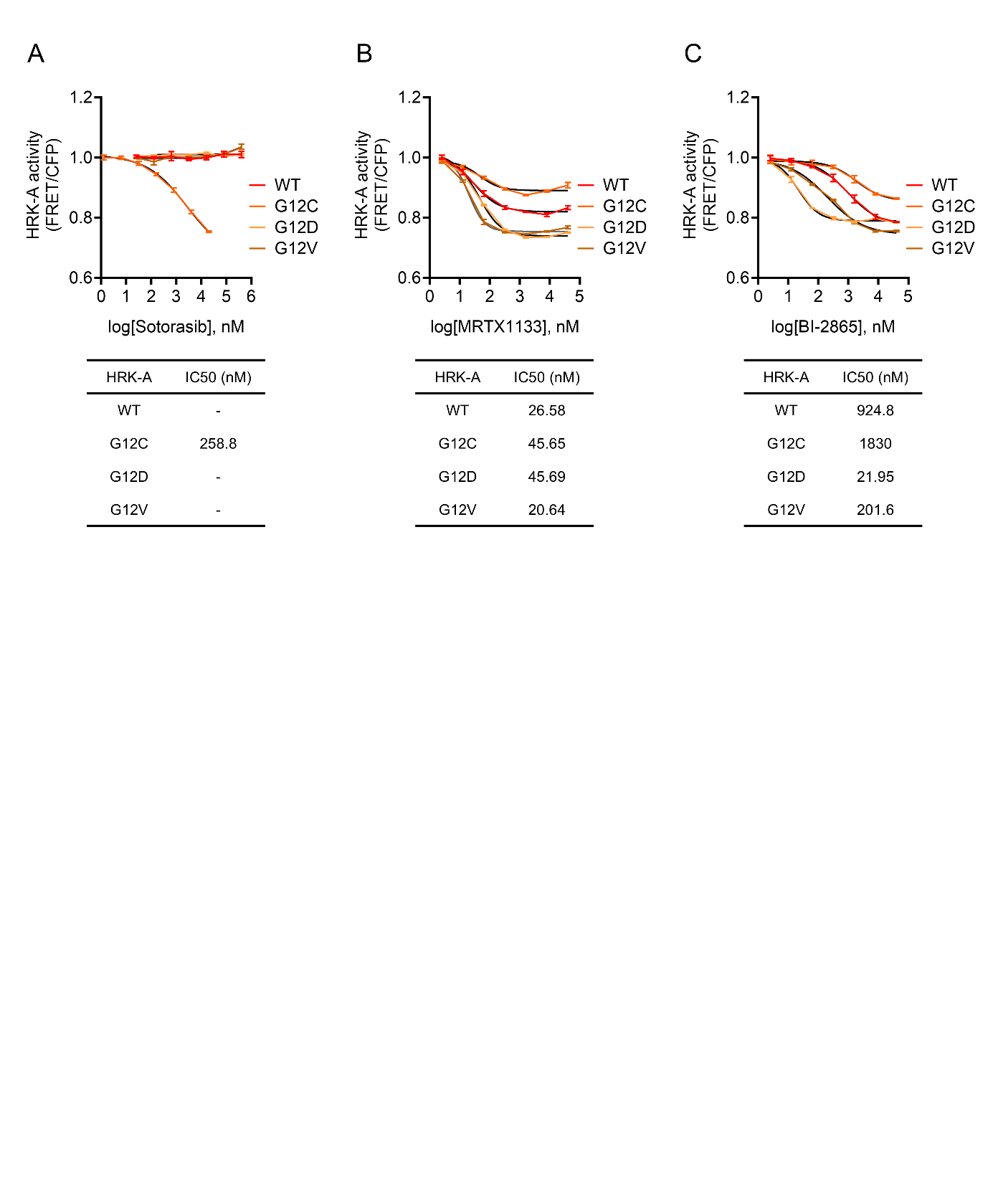


**Figure S9. In vitro dose-response curves and IC_50_ values for KRAS inhibitors measured with HRK-A.** (A) Dose-response inhibition by sotorasib (n = 4) measured as HRK-A activity (FRET/CFP) in an in vitro assay using the indicated KRAS variants. (B) Dose-response inhibition by MRTX1133 (n = 4) measured as HRK-A activity (FRET/CFP) in an in vitro assay using the indicated KRAS variants. (C) Dose-response inhibition by BI-2865 (n = 4) measured as HRK-A activity (FRET/CFP) in an in vitro assay using the indicated KRAS variants. IC_50_ values were calculated by nonlinear regression and are summarized in the tables below each panel.


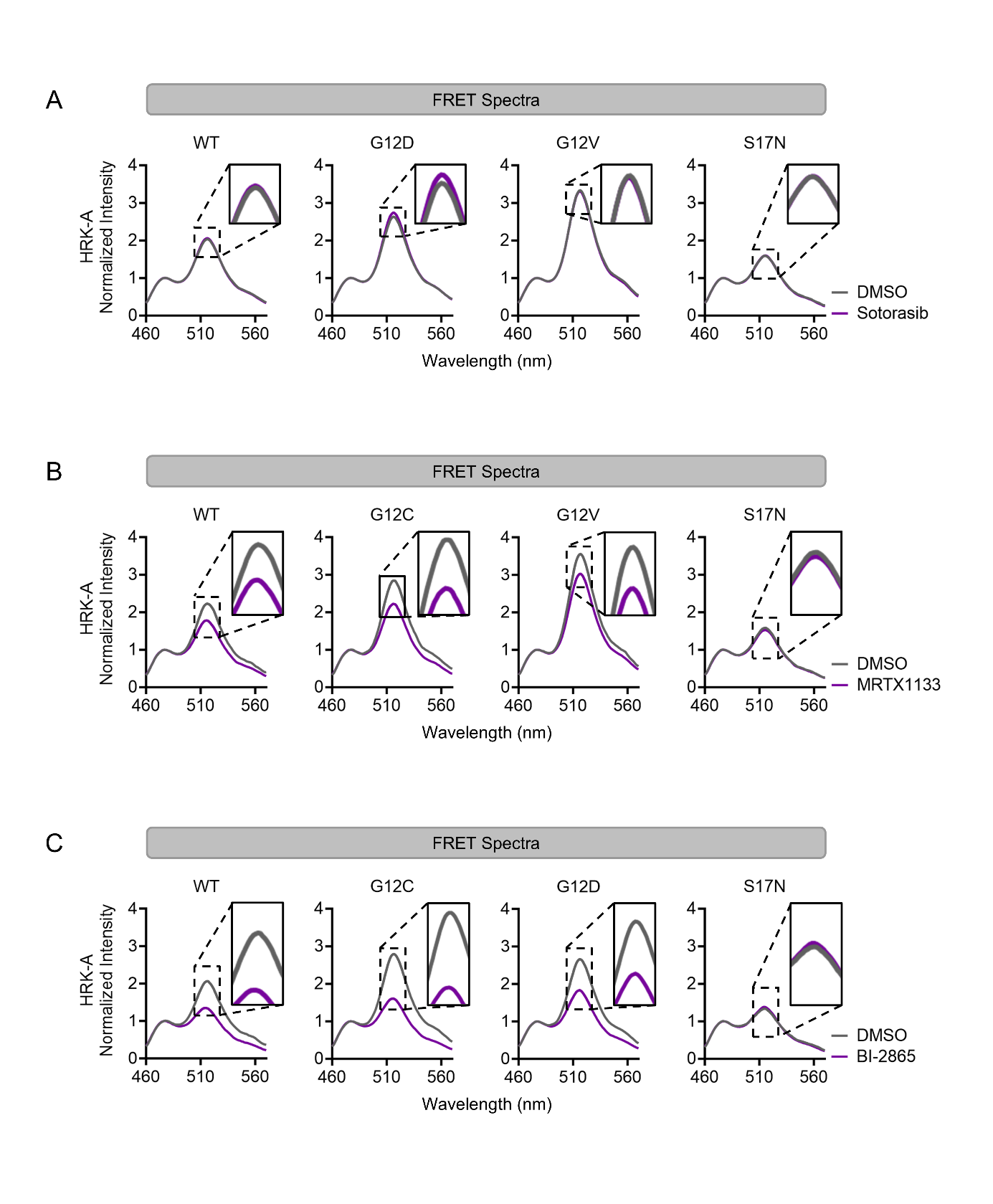


**Figure S10. Spectral FRET analysis of KRAS inhibitor responses across KRAS variants.** (A) Normalized FRET emission spectra of HRK-A variants in Lenti-X 293T cells treated with 500 nM sotorasib or DMSO (0.5% v/v, control) for 24 hours (n = 3). (B) Normalized FRET emission spectra of HRK-A variants in Lenti-X 293T cells treated with 100 nM MRTX1133 or DMSO (0.5% v/v, control) for 24 hours (n = 4). (C) Normalized FRET emission spectra of HRK-A variants in Lenti-X 293T cells treated with 100 nM BI-2865 or DMSO (0.5% v/v, control) for 24 hours (n = 3).


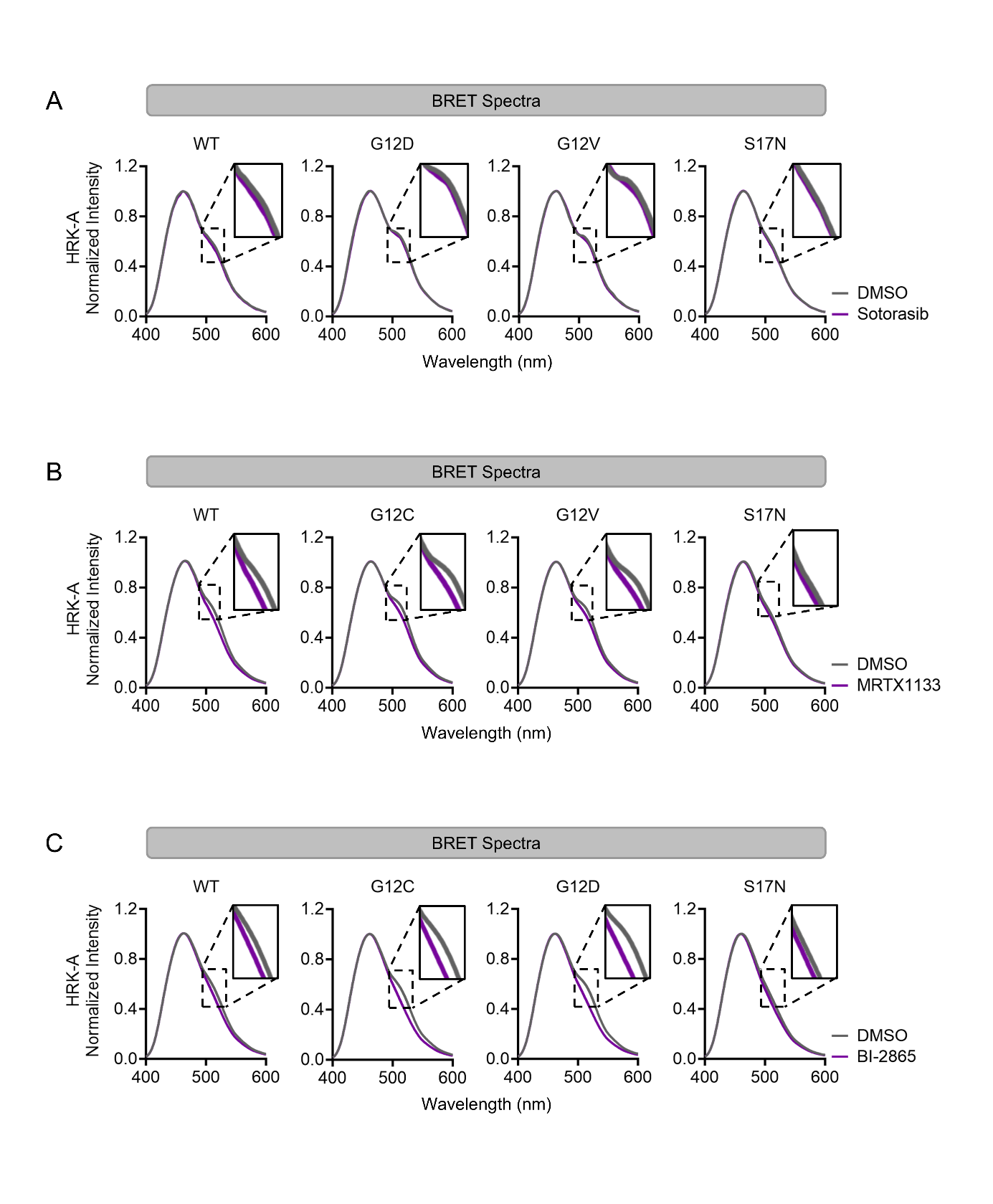


**Figure S11. Spectral BRET analysis of KRAS inhibitor responses across KRAS variants.** (A) Normalized BRET emission spectra of HRK-A variants in Lenti-X 293T cells treated with 500 nM sotorasib or DMSO (0.5% v/v, control) for 24 hours (n = 4-7). (B) Normalized BRET emission spectra of HRK-A variants in Lenti-X 293T cells treated with 100 nM MRTX1133 or DMSO (0.5% v/v, control) for 24 hours (n = 3-5). (C) Normalized BRET emission spectra of HRK-A variants in Lenti-X 293T cells treated with 100 nM BI-2865 or DMSO (0.5% v/v, control) for 24 hours (n = 3).
